# Camera collars reveal macronutrient balancing in free‐ranging male moose during summer

**DOI:** 10.1002/ece3.70192

**Published:** 2024-08-16

**Authors:** Robert Spitzer, Monica Ericson, Annika M. Felton, Morten Heim, David Raubenheimer, Erling J. Solberg, Hilde K. Wam, Christer M. Rolandsen

**Affiliations:** ^1^ Department of Wildlife, Fish and Environmental Studies Swedish University of Agricultural Sciences Umeå Sweden; ^2^ Skogsforsk Uppsala Sweden; ^3^ Faculty of Forest Sciences, Southern Swedish Forest Research Centre Swedish University of Agricultural Sciences Lomma Sweden; ^4^ Norwegian Institute for Nature Research Trondheim Norway; ^5^ Charles Perkins Centre, and School of Life and Environmental Sciences University of Sydney Camperdown New South Wales Australia; ^6^ Department of Wildlife and Rangelands NIBIO Ås Norway

**Keywords:** cervid, deer, large herbivore, macronutrient balancing, nutritional ecology

## Abstract

Understanding how the nutritional properties of food resources drive foraging choices is important for the management and conservation of wildlife populations. For moose (*Alces alces*), recent experimental and observational studies during the winter have shown macronutrient balancing between available protein (AP) and highly metabolizable macronutrients (total non‐structural carbohydrates [TNC] and lipids). Here, we combined the use of continuous‐recording camera collars with plant nutrient analyses and forage availability measurements to obtain a detailed insight into the food and nutritional choices of three wild moose in Norway over a 5‐day period in summer. We found that moose derived their macronutrient energy primarily from carbohydrates (74.2%), followed by protein (13.1%), and lipids (12.7%). Diets were dominated by deciduous tree browse (71%). Willows (*Salix* spp.) were selected for and constituted 51% of the average diet. Moose consumed 25 different food items during the study period of which 9 comprised 95% of the diet. Moose tightly regulated their intake of protein to highly metabolizable macronutrients (AP:TNC + lipids) to a ratio of 1:2.7 (0.37 ± 0.002SD). They did this by feeding on foods that most closely matched the target macronutrient ratio such as *Salix* spp., or by combining nutritionally imbalanced foods (complementary feeding) in a non‐random manner that minimized deviations from the intake target. The observed patterns of macronutrient balancing aligned well with the findings of winter studies. Differential feeding on nutritionally balanced downy birch (*Betula pubescens*) leaves versus imbalanced twigs+leaves across moose individuals indicated that macronutrient balancing may occur on as fine a scale as foraging bites on a single plant species. Utilized forages generally met the suggested requirement thresholds for the minerals calcium, phosphorus, copper, molybdenum, and magnesium but tended to be low in sodium. Our findings offer new insights into the foraging behavior of a model species in ungulate nutritional ecology and contribute to informed decision‐making in wildlife and forest management.

## Graphical abstract

Understanding how the nutritional properties of food resources influence foraging decisions is crucial for wildlife management and conservation. Using a combination of camera collars with plant nutrient analyses and forage availability measurements, we show that moose used macronutrient balancing for tightly regulating their intake of protein to highly metabolizable macronutrients (non‐structural carbohydrates and lipids) during summer. Our findings provide novel contributions to ungulate nutritional ecology and inform decision‐making in wildlife and forest management.

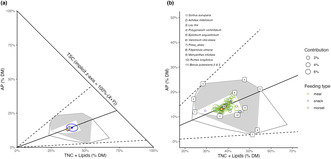

## 1 INTRODUCTION

There is a growing body of evidence that foraging in a wide variety of animals from ants (Krabbe et al., 2019) to primates (Felton et al., 2009) is driven by the goal of reaching a particular target balance of the macronutrients protein, carbohydrates, and fat (Raubenheimer et al., 2022). Reaching a nutritional target, however, is not a straightforward task. Herbivores in particular are often faced with a large number of potential food plants, which contain macronutrients in sometimes widely divergent proportions and are subject to substantial inter‐ and intraspecific variation (Hjeljord et al., 1982), especially in high‐latitude regions with pronounced seasonality. In consequence, nutrient balancing is usually achieved by consuming foods that either are already nutritionally balanced with respect to an organism's nutritional target or through feeding on a variety of nutritionally complementary foods, that is, foods that are individually imbalanced but in the right combination still result in a nutritionally balanced diet (Felton et al., 2016).

The concept of this multidimensional nutritional niche, which encompasses nutritional but also structural characteristics of foods, has expanded the traditional view of organisms falling along a gradient of food generalists to food specialists based on the range of foods consumed (Coogan et al., 2018; Machovsky‐Capuska et al., 2016). A diet that varies broadly in nutritional composition would indicate a food composition generalist, whereas animals restricted to a narrow range of nutritional intakes are food composition specialists. Understanding an animal's nutritional target within the multidimensional nutritional niche framework allows for better predictions of foraging behavior and thus improved management of animal populations and their foodscapes (Felton et al., 2016).

The moose (*Alces alces*) is a large mammalian herbivore in the boreal and boreo‐nemoral forests of the Northern Hemisphere and a major driver of the functioning of these ecosystems (Pastor et al., 1988). Moose are classified as browsers (sensu Hofmann (1989)) with a gut morphology specialized for the digestion of twigs, leaves, and forbs but poorly adapted for digesting graminoids (Clauss et al., 2010). The ability of moose to consume large quantities of coniferous and deciduous tree browse frequently places moose at the center of human–wildlife conflicts over browsing impacts on commercial tree species (Linnell et al., 2020). At the same time, moose are a highly valued game species (Jensen et al., 2020) and moose management is thus faced with the challenge of maintaining healthy, harvestable populations while minimizing conflicts with forestry (Dressel et al., 2018; Timmermann & Rodgers, 2015).

Several nutritional influences on foraging choices of moose and other cervids have been suggested, including minerals (Robbins, 1994), plant secondary metabolites (Freeland & Janzen, 1974), energy (Belovsky, 1978; Schoener, 1971), or protein (Mattson, 1980). While each of these food constituents is important, there has been little evidence for the maximization or limitation of any single constituent being the main driver of foraging choices in northern cervids (Felton et al., 2018). Instead, it is becoming increasingly evident that foraging involves balancing multiple nutrients simultaneously (Simpson & Raubenheimer, 2012).

For moose, this nutrient balancing hypothesis was first tested in controlled winter‐feeding trials with captive animals, which revealed that moose selected the foods on offer to reach a nutritional target resembling the protein to non‐protein energy ratio of willow twigs (*Salix* spp., hereafter simply referred to as *Salix*; Felton et al., 2016). A large‐scale study using rumen content analysis of free‐ranging moose in Sweden further refined the picture by showing that moose regulated their diets to maintain a tight relationship between protein and highly metabolizable macronutrients (non‐structural carbohydrates and lipids), despite broad differences in eaten and available foods (Felton et al., 2021). Similarly, Ma et al. (2019) reported that moose maintained a specific ratio of protein to total non‐structural carbohydrates in their diets across six populations in China, despite differences in forage availability. More specifically, Spitzer et al. (2023) showed that moose used complementary feeding on Scots pine (*Pinus sylvestris*) and ericaceous shrubs to achieve a ratio of protein to total digestible carbohydrate similar to *Salix* browse when the latter was not abundantly available. The above studies all focused on the winter season and, except for the feeding trial, relied on reconstructed diets from proxies such as rumen content and feces.

**FIGURE 1:**
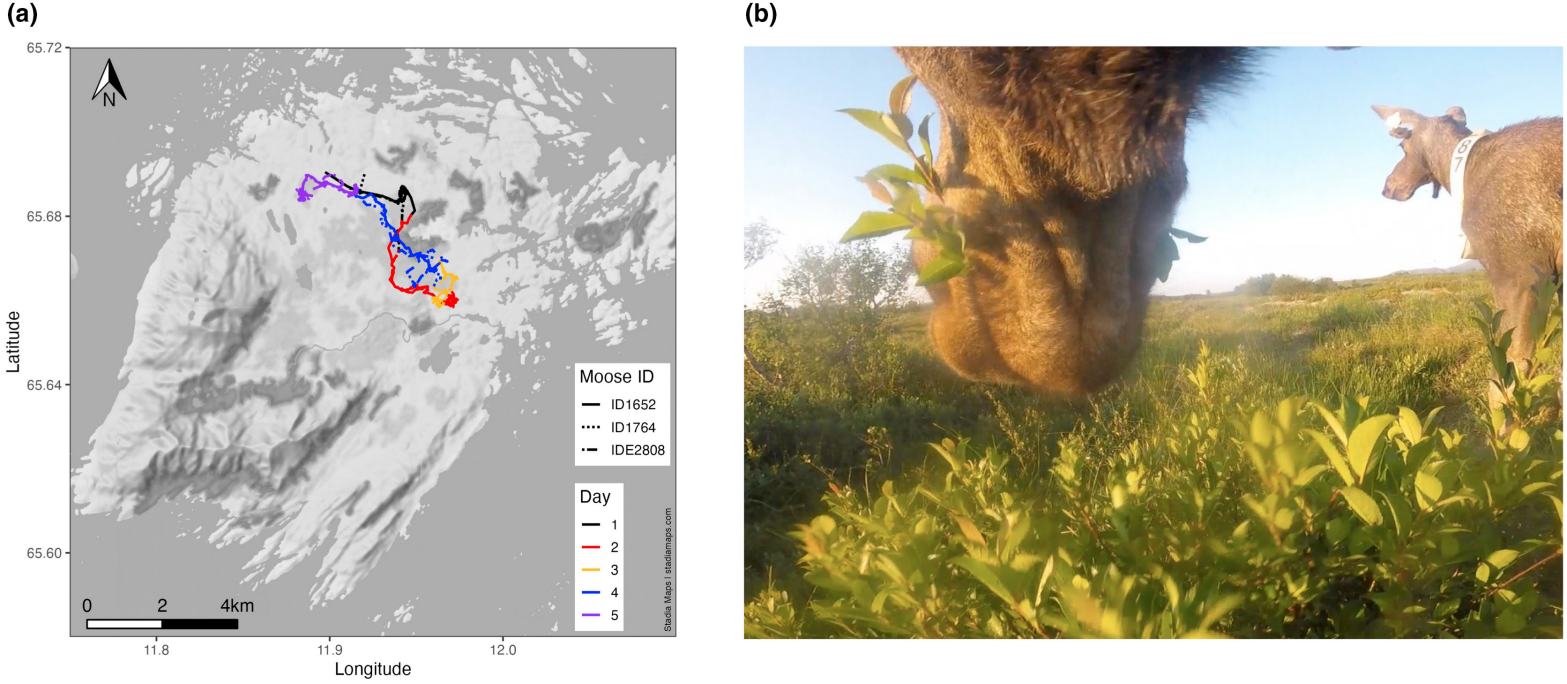
(a) Map of the study area on Vega Island, Norway. The routes walked by the three camera‐collared moose during the 5‐day observation period are shown as different line types with the daily sections being indicated by different colors. (b) Video still from a collar camera showing a collared moose feeding on *Salix* sp. on Vega Island during July 2022. To the right is another individual, equipped with a GPS collar without camera.

In the present study, we combined the use of continuous‐recording camera collars with plant nutrient analyses and forage availability measurements to obtain a detailed insight into the food and nutritional choices of three wild moose in Norway over a five‐day period in summer (July). In summer, available forage plants are at their greatest diversity and abundance, which makes it reasonable to assume that observed diets approximate the nutritional target. Due to the high northern latitude, the study area (Vega Island, Figure 1) experiences 24 h of daylight in July, allowing for high‐quality video footage at any time of the day. The study area also did not contain any large predators whose presence could have influenced moose habitat choice and thereby access to forage (via ‘landscape of fear’ effects, e.g., Churski et al. (2021)), nor were moose hunted during the study period. Much knowledge can be gained regarding a species' nutritional geometry by closely following even a small number of individuals as was demonstrated, for example, by Johnson et al. (2013) in the case of a single baboon female.

The combination of high‐temporal resolution foraging observation, plant nutrient analyses, and forage availability measurements enabled us to (i) characterize moose diets botanically and nutritionally, (ii) test for macronutrient balancing during summer and compare results to the reported patterns for winter diets, (iii) test for evidence that moose achieve macronutrient balancing by complementary feeding (i.e., the deliberate mixing of plant species and plant parts to reach their nutritional target), and (iv) assess moose foraging in the context of several minerals, which have been reported as important for ruminants, namely, sodium (Na), calcium (Ca), phosphorus (P), copper (Cu), molybdenum (Mo), and magnesium (Mg) (see Box 1 for details). Minerals are often lacking in studies of large herbivore foraging but may nevertheless be highly influential in foraging decisions (Felton et al., 2018; Wam et al., 2018).

BOX 1 **Dietary minerals**

**Sodium (Na)**

Sodium is an essential mineral for regulating fluid and electrolyte balance in the body and important for nerve functioning (Strazzullo & Leclercq, 2014). Sodium concentrations in natural moose forages are usually well below the suggested threshold of 0.1% or more of dry matter (DM) intake for domestic ruminants (Botkin et al., 1973). An exception are aquatic macrophytes, which are often high in sodium content, and whose use by moose has been well documented (Belovsky & Jordan, 1981; Faber et al., 1988; Fraser et al., 1984).

**Calcium (Ca) and Phosphorus (P)**

Calcium is the most abundant mineral in the animal body and, together with phosphorus, is crucial for the development of bone structures (McDonald, 2011). A Ca:P ratio within the range of 1:1 to 2:1 is considered suitable for livestock, but higher ratios of up to 7:1 can be tolerated if phosphorus requirements are met (McDonald, 2011; Ricketts et al., 1970; Wise et al., 1963). Suggested minimum requirements for calcium and phosphorus in ruminant diets have been reported as 0.2% DM (Ohlson & Staaland, 2001).

**Copper (Cu) and Molybdenum (Mo)**

Copper is important for the absorption of iron, plays a role in several enzymatic systems, and is necessary for pigmentation (McDonald, 2011). Molybdenum is mostly involved in enzymatic processes, and dietary requirements are extremely low. High levels of molybdenum in the diet can lead to copper deficiency. Toxic problems in ruminants can occur under conditions of high molybdenum concentrations (>20 PPM), absolute copper deficiency (<5 PPM), or low Cu:Mo ratios (≤2:1) (Auza et al., 2002).

**Magnesium (Mg)**

Magnesium is a common enzyme activator and a key element in cellular biochemistry and function (McDonald, 2011). We could not find any studies on the magnesium requirements of moose, but dietary recommendations for dairy and beef cattle typically range from 0.12% to 0.3% DM (Pinotti et al., 2021).

Extending the study of macronutrient balancing to the summer improves our understanding of how generalizable the observed patterns may be for moose and potentially other wild ruminants. This is crucial for enhancing our ability to make informed decisions regarding wildlife and forest management strategies toward promoting the long‐term sustainability of moose populations and their habitats.

## 2 MATERIALS AND METHODS

### 2.1 Study area

Data were collected on the island of Vega (119 km^2^, 65°40′ N, 11°55′ E) in Nordland County, northern Norway (Figure 1). The island is dominated by moorland and marshes (44%) interspersed with forests (15%) of predominantly Norway spruce (*Picea abies*), Sitka spruce (*P. sitchensis*, introduced), *P. sylvestris*, and birch (*Betula* spp.), as well as farm and grasslands (21%). Three quarters of the island lie below 80 m a.s.l. but steep mountains rise to an elevation of 800 m in the southwestern part of the island (Angeloff et al., 2004). The climate is oceanic with temperatures of 13°C ± 3 SD during July and August. Precipitation is approximately 240 mm during the summer (Herfindal et al., 2009). Moose colonized the island in 1985 by swimming from the mainland. Moose hunting started in 1989 and has since 1992, when a long‐term moose research project by the Norwegian authorities started, kept the breeding population at a size between 24 and 54 individuals (Solberg et al., 2024).

### 2.2 Data collection

#### 2.2.1 Moose collaring and camera programming

Three male moose (ID: 1652,1764, E2808) between 2 and 7 years of age were captured during January 2022 as part of the moose research program on Vega (permit 21/243943: www.mattilsynet.no/dyr/forsoksdyr/soknader?id=28717) and outfitted with camera collars (VERTEX PLUS‐IRIDUM with 7D Battery and Vertex PLUS Camera Option 238 GB; Vectronic Aerospace GmbH). As funding limited us to three individuals, we chose to collar only males to control for possible effects of sex on diet and nutrient balancing in general, and for the unforeseeable effects of nursing calves on females specifically. The data storage capacity of the camera collars limited our near‐continuous video recording to an observation period of 5 consecutive days. However, results from a moose feeding trial preliminarily suggested that moose quickly sense changes to the macronutritional conditions within their gastrointestinal tract, and adjust their food choices accordingly within 30–60 h (Felton et al., 2016).

Cameras were programmed to record 25 s of video every 3 min during the study period from July 1, 2022, at 00:00:37 to July 5, 2022, at 23:58:38, resulting in approximately 16 h of video footage per collar (currently the maximum video data storage capacity). Additionally, GPS coordinate fixes were transmitted at 6‐min intervals during the study period. Video recordings could not be accessed remotely. Collars were therefore remotely released on July 6, 2022, using a Vectronic UHF release transmitter and retrieved from the field. The video recordings were then manually downloaded for analysis.

#### 2.2.2 Video analysis and diet composition

Videos were manually viewed and analyzed. We distinguished six behavior states (feeding, moving, ruminating, lying, standing, and other) and quantified each as seconds of observation time. For each feeding observation, we recorded which plant was eaten and for how long (in sec), and also which part of a plant was consumed, that is, the whole plant (for non‐woody forages), leaves stripped off twigs (hereafter ‘stripped leaves’), or twigs+leaves ingested together (woody forages). Like other deer, moose lack upper incisors. However, their dexterous lips and tongue make them especially skilled at selectively stripping leaves from twigs when they choose to do so. When biting off twigs, they tear them between their lower incisors and a hard upper palate, resulting in ragged ends. Consumed plant species that could not be identified to species level in the videos were classified to the lowest possible taxonomic level, typically genus.

We further classified feeding observations into long feeding bouts dubbed ‘meals’ (observations of feeding across > than 3 consecutive videos and separated by > than 3 consecutive videos without feeding observations), shorter feeding bouts, that is, ‘snacks’ (observations of feeding across 1–3 consecutive videos with a minimum of 5 s of feeding in at least one video, separated by >3 consecutive videos from other feeding bouts), and ‘morsels’ (isolated bites on single food items, <5 s of feeding time and not part of a ‘meal’ or ‘snack’).

We quantified botanical diet compositions by converting the observed feeding times per food item to proportions, assuming approximately equal intake rates in terms of grams ingested/second. For converting the botanical diet composition to nutritional compositions, we first adjusted them to dry matter proportions and then multiplied the proportion of each food item in a diet by its dry matter‐based nutritional content (Felton et al., 2016; Ma et al., 2019). If leaves and twigs were consumed together, we averaged the nutritional values for leaves and twigs of the respective plant species.

For the conversion of macronutrients to metabolizable energy, we used the conversion factors of 9 kcal/g for lipids and 4 kcal/g for proteins and carbohydrates (Hecker et al., 2021; Merrill & Watt, 1973; Shrestha et al., 2020). The resulting energy values for each macronutrient fraction in each diet or food item were then converted to percentages by dividing the energy value for each macronutrient by the sum of the energy values for all macronutrients, that is, the total metabolizable energy contained in a diet or food item (Shrestha et al., 2020).

#### 2.2.3 Forage availability

To measure forage availability, we retraced the routes walked by each moose during the observation period directly after retrieval of the collars based on the GPS locations. Following Spitzer et al. (2021), we used the step‐point method (Coulloudon et al., 1999; Evans & Love, 1957) to record vegetation hits on a pole (i.e., every plant touching the pole was counted as one hit) within the summer browsing height range of moose (0–2.8 m; Nichols et al. (2015)) approximately every 25 m along the routes. Such vegetation hits are analogous to potential browsing bites and can be converted into proportions. Those can then be compared to the proportions of the corresponding food items in the diet as a measure of selectivity.

However, not all vegetation present in the environment should be considered available to moose (e.g., some plants may be toxic or indigestible). Inclusion of such plants in availability measurements dilutes the proportion of actual forages and could thereby inflate selectivity results. For a more conservative estimate, we therefore later only used the hits on plants that moose were observed eating at least once in the videos to quantify forage availability in our analyses.

#### 2.2.4 Plant sample collection and nutrient analyses

Samples of putative moose forage plants were collected alongside the retraced moose routes. Because the sample collection started before the video data analyses were completed, we initially had to rely on a priori knowledge about moose diets from local personal communication and the literature. For the suggested main food items, that is, downy birch (*Betula pubescens*; hereafter simply ‘birch’), *Salix*, rowan (*Sorbus aucuparia*), European aspen (*Populus tremula*), and meadowsweet (*Filipendula ulmaria*), we aimed at collecting 10 samples (2 from each area used per day by moose during the 5‐day observation period).

During each of the 5 days, the moose traversed similar, frequently overlapping daily routes (Figure 1). We therefore randomly chose one individual's route each day for the vegetation collections. To further randomize collections, we collected the fresh plant material (approximately 150 g) at the first two instances the respective food items were encountered along the route the moose had walked. Sample collection emulated moose browsing, and twig diameter for tree species was kept at ≤4 mm to keep a consistent cutoff. This is assumed to be the optimal diameter of birch twigs for moose in winter regarding energy maximization (Vivås et al., 1991). No similar diameter studies from summer were available. For deciduous tree species, we separated leaves and twigs. Twigs were cut from the top 20 cm of shoots, from shoots available within the browsing height, whereas forbs, graminoids, and other herbaceous vegetation were collected whole (i.e., cut close to the ground). From woody dwarf shrubs such as *Vaccinium* spp., we sampled ‘mouthfuls’, that is, the upper 10–20 cm of shoots.

In addition to the putative main food items, we collected at least one sample of plant species that we considered likely to be food items. Prior to the conclusion of the field work, we rapidly screened the video footage from the collar cameras to identify foraged plants we had not yet collected and then returned to the field to obtain samples of those missing species. During the rapid screening, we missed a single feeding observation on *P. abies* and thus collected no sample in the study area. Instead, we used a sample from Sweden collected at the same latitude in the analyses. In a few instances when moose were rapidly feeding on mixed, low vegetation in meadows or ley fields, we could not identify the ingested plant species in the videos. We therefore randomly collected handfuls of vegetation from these two habitats and referred to them as ‘meadow‐mix’ and ‘ley‐mix’ in the analyses. Ley fields were similar to meadows but consisted mostly of grasses grown for intensive agricultural harvesting, whereas meadows contained a more diverse mix of grasses, wildflowers, and other forbs. After collection, the plant samples were kept frozen at −20°C until further processing.

To determine water content, we weighted the samples before and after oven drying at 60°C until a constant weight was reached (typically within 48 h). We then ground the dried samples using a laboratory cutting mill with a 1‐mm sieve (Krizsan & Huhtanen, 2013). For each species, we pooled the ground material to incorporate the between individual and between sampling location variation in nutrient content (Felton et al., 2021; Spitzer et al., 2023). The chemical analyses for nutrient content were performed by the DairyOne Forage laboratory (USA, www.dairyone.com). The nutritional components analyzed for this study and methods of analysis are provided as Table S1.

Following Spitzer et al. (2023), we calculated crude protein (CP) as N*6.25, available protein (AP) as CP‐ADICP (acid detergent insoluble crude protein), cellulose as acid detergent fiber (ADF) – lignin, hemicellulose as neutral detergent fiber (aNDF) – ADF, and total non‐structural carbohydrates (TNC) as water‐soluble carbohydrates (WSC) + starch. Total metabolizable structural carbohydrates (cellulose + hemicellulose) were denoted as TSC.

### 2.3 Statistical analyses

#### 2.3.1 Nutrient balancing

To investigate patterns of nutrient balancing, we used right‐angled mixture triangles (RMTs; Raubenheimer (2011)) to plot the compositions of forage plants and observed moose diets as Cartesian points within the RMTs based on either metabolizable energy content or macronutrient proportions (Aryal et al., 2015; Felton et al., 2021; Shrestha et al., 2020). For the latter, we used the same RMT dimensions as Felton et al. (2021), namely, highly metabolizable macronutrients (TNC and lipids; *x*‐axis), available protein (*y*‐axis), and TSC (cellulose + hemicellulose) on the implicit *z*‐axis. To visually quantify the realized nutritional niche of moose, we used convex hull polygons and 95% confidence region ellipses around foraged plants (Shrestha et al., 2020). We considered the macronutrient composition of the average observed diet across individuals and the observation period to be the intake target.

We used nutritional rails within the RMTs to illustrate specific ratios of macronutrients and for comparison with previously reported nutritional targets for moose such as the protein to non‐protein macronutrient ratio of willow twigs (Felton et al., 2016), and the tight relationship between AP and TNC + lipids in moose rumens (Felton et al., 2021). Nutritional rails are a concept within the Geometric Framework for Nutrition (GFN, for a full description, see Raubenheimer et al. (2009) and Simpson and Raubenheimer (2012)). In brief, nutritional rails of various food types correspond to radials extending outwards from the origin of a graph with their slopes representing the ratio of the macronutrients represented by the respective axes. Foods whose nutritional rails intersect the intake target of an organism (moose in our study) are considered nutritionally balanced for this organism. Foods whose rails fall on opposite sides of the intake target are considered to be nutritionally complementary with respect to the intake target in the dimensions of the GFN because they allow an animal to reach its nutritional target by mixing the intake of those individually imbalanced foods. We considered food items as nutritionally balanced for moose if their AP:TNC + lipids ratio fell within one standard deviation of the nutritional target.

#### 2.3.2 Foraging niche and selectivity

To quantify dietary niche overlap between the three moose individuals, we used Pianka's index (Pianka, 1988) on the botanical and nutritional diet compositions. The index ranges from 0 (no overlap) to 1 (complete overlap). We used Pearson correlations to test the relationships of macronutrients in forages and observed diets. For comparing utilized forages to their availability (i.e., selectivity), we used a scatterplot with 1:1 line and the Spearman rank correlation (to account for non‐normality of the data) for testing the relationship.

#### 2.3.3 Complementary feeding

To investigate complementary feeding, we compared the observed diets as if composed of only the nutritionally imbalanced (and potentially complementary) foods, that is, food items that individually did not meet the moose nutritional target. Food items meeting the nutritional target such as *Salix* were computationally removed from the foraging data. We then used autocorrelation analysis to investigate patterns of complementary feeding over subsequent feeding events in relation to the target ratio of macronutrients. In this context, a complementary feeding event was defined as feeding observations of one nutritionally imbalanced food item (i.e., a food item whose nutritional rail fell above or below the nutritional target) before switching to the next imbalanced food item. Significance of autocorrelation of the macronutritional ratios in these complementary feeding events was tested using Durban–Watson tests from R package ‘car’ (Fox & Weisberg, 2019). The test statistic for the Durbin–Watson test ranges from 0 to 4 with values >2 indicating negative autocorrelation and values <2 indicating positive autocorrelation. A negative autocorrelation implies complementary feeding (feeding on an item below the target rail to an item above it, or vice versa). To further test for non‐randomness of the observed complementary feeding patterns, we also simulated random complementary feeding by randomly drawing complementary food items in a sequence corresponding to the average number of observed complementary feeding events across the three moose. We repeated this procedure 1000 times and tested each simulated feeding sequence for autocorrelation in the same way as the observed complementary feeding sequences.

To compare the means of the macronutrient ratios in observed and simulated complementary feeding sequences, we used Welch's *t*‐test and Levene's test for testing equality of variances. To equalize sample sizes for the latter two tests, we randomly drew three feeding sequences (to match the observed feeding sequences across the three moose individuals) 1000 times from the simulated data before testing, and therefore report the means of the test statistics and *p*‐values for these tests. All statistical analyses were performed in R version 4.2.1 (R Core Team, 2022) with a significance threshold of alpha = 0.05.

## 3 RESULTS

**FIGURE 2:**
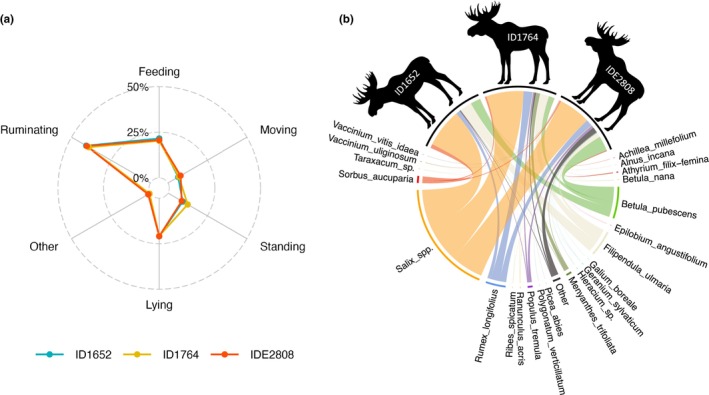
(a) Spider plot of different behavioral states (as percentages of total observation time) for three moose individuals on the island of Vega, Norway, for 5 days in July 2022 with 24 h of daylight (individuals indicated by colors and ID). The behaviors were recorded with camera collars on the animals. (b) Chord diagram of the average botanical diet composition of the moose during these days. The average diet composition for each individual is indicated by the width of the differently colored chords below the black circle segments, with chords linking to the corresponding food item at the opposite end. The widths of the colored circle segments correspond to the proportions of the respective taxa in the diet, respectively, of individuals or pooled (i.e., the average across all three individuals).

The three video collars yielded a total of 6422 videos of which 6399 (99.6%) were of interpretable quality; 23 video files (0.4%) were corrupted for unknown reasons. Overall activity patterns among the three individual moose were very similar (Figure 2a) with approximately 25% of the observation time dedicated to feeding. Activity patterns across the hours of the day were similar to the overall pattern across the study period with no distinct peaks for feeding (Figure S1).

During the 5‐day observation period, the three moose utilized an area of approximately 13.25 km^2^ in the northcentral lowlands of the island. For the first two observation days, they moved toward the southeast before looping back to the northwest on day four, returning almost to the starting point of the observations on day five (Figure 1). The mean altitude a.s.l. of their route was 15 m ± 8 SD.

Of 42 collected putative food items, 25 were eaten by moose (19 identified to plant species level, 3 to genus level, and 2 corresponding to plant mixtures [meadow‐mix and ley‐mix]; Table S2a). For all woody forages except birch, moose consumed twigs and leaves together. Birch therefore corresponded to two food items (stripped leaves and leaves + twigs). Diets were dominated by only a few different foods (Figure 2b) with *Salix* contributing the most (51%) to the average diet (Table S2a). Together, the nine most eaten food items (*Salix*, birch stripped leaves, birch leaves + twigs, *F. ulmaria*, *Sorbus aucuparia*, *P. tremula*, dock (*Rumex longifolius*), bogbean (*Menyanthes trifoliata*), and meadow‐mix) comprised at least 95% of individual diets and 97% of the overall average diet.

Feeding was observed in 1953 instances (ranging from 629 to 666 per individual), corresponding to 32–37 meals, 8–14 snacks (shorter than a meal, see definition in methods) and 1–7 morsels (isolated bites not part of a meal or snack) across the three individuals. Mean dietary niche overlap (Pianka's index) between the three individuals was high; 0.94 ± 0.03 SD for the botanical diet compositions, and 0.99 ± 0.0001 SD for the macronutritional compositions.

**FIGURE 3:**
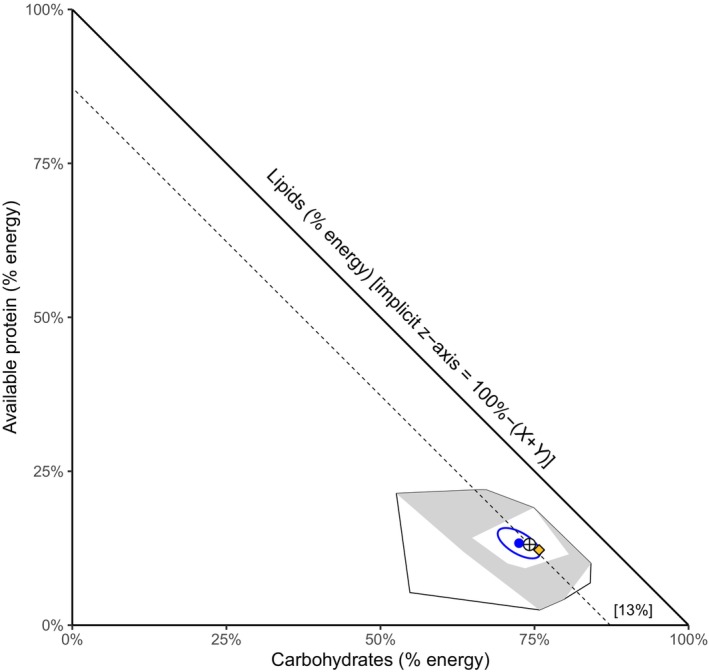
Right‐angled mixture triangle showing the relative proportions of metabolizable energy from carbohydrates (TNC + TSC), protein, and lipids of forages on a dry matter basis, as used by camera‐collared moose on the island Vega in Norway in July 2022. Lipids are shown on the implicit *z*‐axis with values increasing toward the origin. For example, the dashed line shows the percentage contribution of lipid energy to the average moose diet (13%). The black polygon corresponds to the nutrient space demarcated by 42 different putative food items along complete moose routes for five consecutive days. The area shaded in gray indicates the nutrient space of the 25 food items that were consumed by moose (the realized food niche) and the white polygon inside the gray encompasses a subset of 9 food items, which together constituted at least 95% of moose diets. The blue dot (with 95% confidence region ellipse) indicates the mean macronutrient energy proportion of the consumed forages and corresponds to what the diet would be if moose had randomly foraged in equal proportions of those foods. The ‘crosshairs’ show the average observed diet across the three moose individuals and the yellow diamond denotes *Salix*, the main food item.

The energy‐based RMT (right‐angled mixture triangle) showed that moose, as expected, derived most energy from carbohydrates ($\bar{x}$ = 74.2% ± 1.1SD) followed by protein ($\bar{x}$ = 13.1% ± 0.1SD) and lipids ($\bar{x}$ = 12.7% ± 1.1SD; Figure 3). This corresponds to a protein energy to non‐protein energy ratio (PE:NPE) of 0.15. The 25 foraged food items ranged from 53% to 84% ($\bar{x}$ = 72.5% ± 7.1SD) in metabolizable energy from carbohydrates, 2.5%–22% from protein ($\bar{x}$ = 13.3% ± 5.1SD), and 6%–26% for lipids ($\bar{x}$ = 14.2% ± 5.6SD). Although the average moose diet was slightly higher in carbohydrate energy and slightly lower in lipid energy than the average of the forages, these differences were not significant based on the 95% confidence region (Figure 3).

**FIGURE 4:**
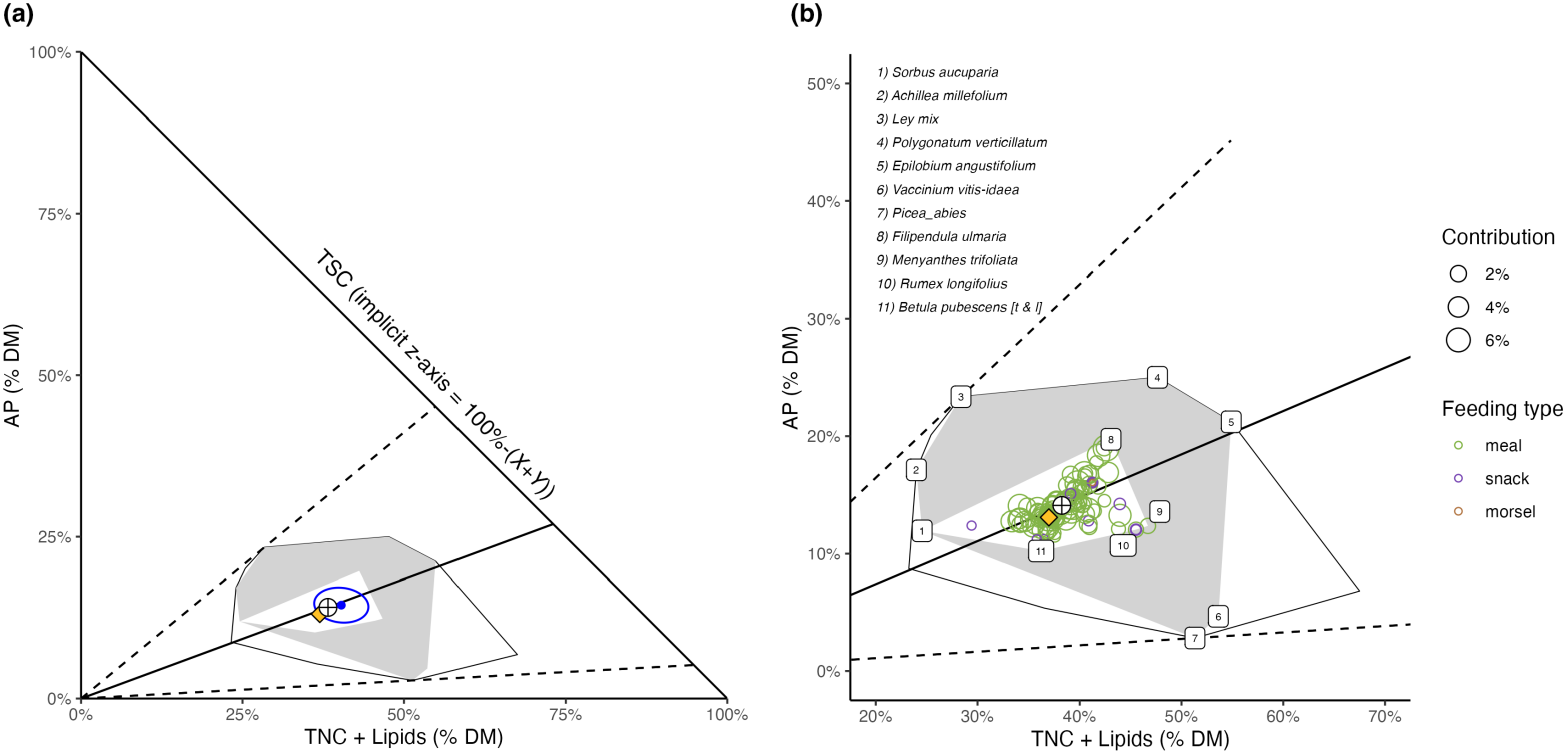
(a) Right‐angled mixture triangle depicting the relative components of macronutrient content in moose diets as percentages of total macronutrients in g dry matter (DM), based on camera‐collared moose on the island Vega in Norway in July 2022; *x*‐axis: Highly digestible energy sources (total non‐structural carbohydrates and lipids (TNC + lipids)), *y*‐axis: Available protein (AP), and the implicit *z*‐axis: Total digestible structural carbohydrates (TSC, cellulose + hemicellulose). The interpretation of the polygons, blue dot, ellipse, crosshairs, and yellow diamond is the same as in Figure 3. The solid black line intersecting the crosshair point corresponds to the AP:TNC + lipids ratio of the intake target. Dashed radials indicate the highest and lowest AP:TNC + lipids ratios among eaten forages. (b) A closer look at the nutrient space utilized by moose with numbers indicating the 11 foods, which delimited the total realized niche (gray polygon, including 25 food items observed to be ingested) and 95% of the diet (white polygon inside the gray). Colored circles indicate different feeding types (meals: Green, snacks: Purple, and morsels: Brown). Their size corresponds to the proportion each feeding type contributed to the total diet across the study period based on observation times.

The macronutrient proportions based on RMTs showed similar results for the diet and the forage (across 25 used food items), that is, the average observed diet fell into the 95% confidence region of forage averages (Figure 4a). The only macronutrient that occurred in significantly lower amounts in the diet ($\bar{x}$ = 18.6% ± 2.6SD) than what would have been expected from eating equal proportions of the forages ($\bar{x}$ = 21.0% ± 5.7SD) was total non‐structural carbohydrates TNC (Welch's *t*‐test, *p* < .05; Figure S2). The average diet across the three moose individuals suggested a nutritional target of 14.1% ± 0.15SD protein, 38.2% ± 0.50SD TNC + lipids, and 47.7% ± 0.63SD total structural carbohydrates TSC, corresponding to an available protein to non‐protein macronutrient ratio (AP:NPM) of 0.16 ± 0.002SD.

The AP:TNC + lipids ratio was 1:2.7 (=0.37 ± 0.002SD). Averaged across all meals (moose individuals pooled) the AP:TNC + lipids ratio was nearly identical but more dispersed (0.36 ± 0.03SD). We used the interval of one standard deviation around this AP:TNC + lipids ratio (0.33–0.39) as a conservative threshold for nutritionally balanced food items. Of the 25 consumed foods, only five met this threshold: *Salix*, birch leaves, fireweed (*Epilobium angustifolium*), dandelion (*Taraxacum* spp.), and bog bilberry (*Vaccinium uliginosum*). Among the 25 consumed foods, the AP:TNC + lipids ratio was highest for ley‐mix (1:1.2) and lowest for *P. abies* (1:18.3). The intake target of the Vega moose resembled the macronutritional composition of *Salix* and meals aligned closely with the nutritional rail of the intake target (Figure 4b).

Macronutrients were strongly correlated in moose diets but not across forages except for TNC (and TNC + lipids) and TSC (all 25 forages pooled; Table 1). AP and TNC + lipids showed the strongest correlation in moose diets (Table 1).

**TABLE 1:** Pearson correlation coefficients between macronutrients in moose diets (available protein (AP), total non‐structural carbohydrates (TNC), total digestible structural carbohydrates (TSC), and lipids (L); upper triangle above the diagonal) and 25 forages (lower triangle below the diagonal, in italics and shaded) on the island Vega in Norway in July 2022.

	AP	TNC	TSC	L	TNC + L
AP	–	.66***	−.41***	−.21**	.67***
TNC	*.1*	–	−.25**	−.49***	.96***
TSC	*.01*	−.42*	–	−.27***	−.36***
L	*.07*	*−.31*	*−.1*	–	−.21**
TNC + L	*.13*	.95***	−.47*	*−.01*	–

*Note*: Significant correlations are marked with asterisks (*p < .05, **p < .01, ***p < .001).

**FIGURE 5:**
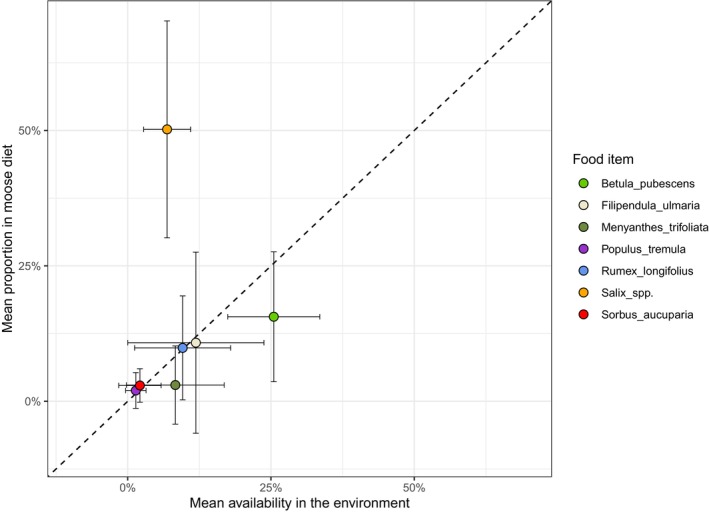
Scatterplot with 1:1 line (dashed) of the relationship between availability and percentage contribution to moose diets of food items. Availability determined by point intersect in field (proportion of hits), and diet determined using camera collars on the animals (proportion of feeding time). For improved visibility, only food items that contributed >1% to the average diet across the study period (5 consecutive days in July 2022, on the island of Vega Norway) are shown. The error bars indicate the standard deviation.

There was no positive correlation between the availability of forages and their proportion in the diet (Spearman's rho = .42, *p* = .123), which suggest that moose did not forage in proportion to food availability. *Salix* was clearly selected for, that is, eaten in much higher proportions than what would have been expected from availability (Figure 5).

**FIGURE 6:**
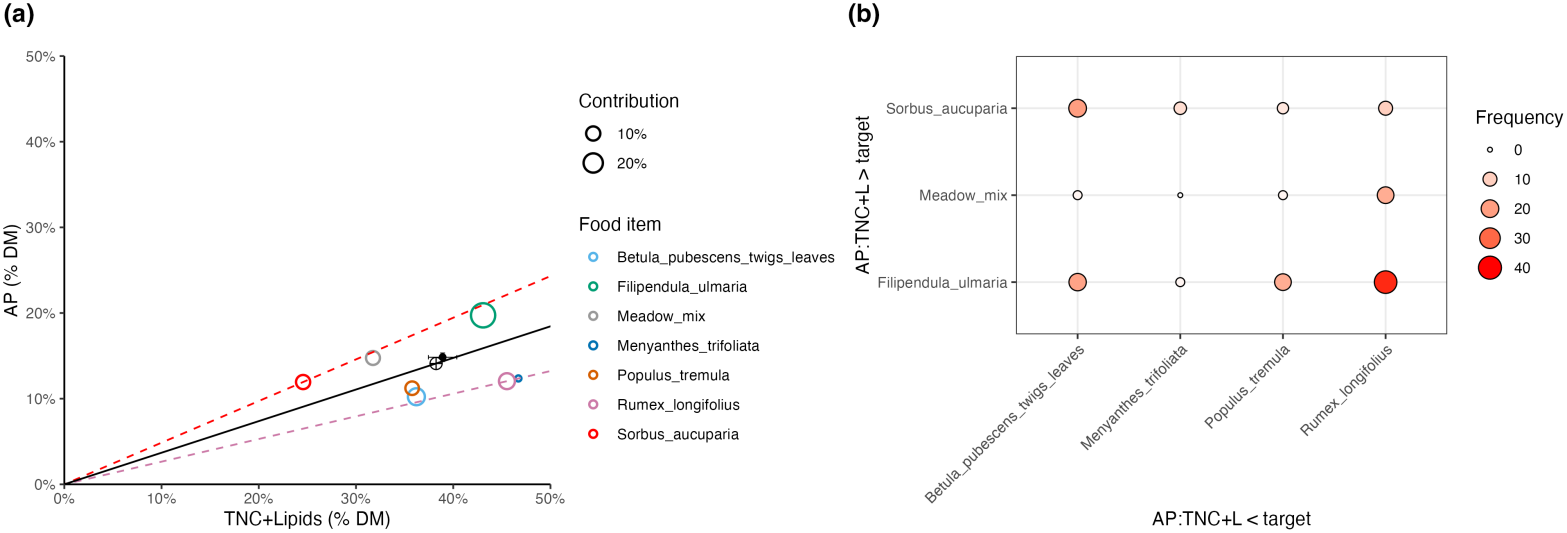
(a) Right‐angled mixture triangle with the solid black dot indicating the average moose diet as if consisting only of imbalanced food items (i.e., with the balanced food items computationally removed) on the island of Vega (Norway) for 5 continuous days in July 2022, in relation to the complete average diet (i.e., the intake target, marked by crosshairs). The error bars denote the standard deviation across the three individuals (very small so difficult to see). Colored circles show the nutritional composition of the complementary food items, which contributed 95% to the diet consisting only of imbalanced food items with their size scaled to their contributions to that diet. Radials depict the macronutrient ratio of the intake target (black) and the ratios of the plotted complementary food items, which were farthest away from the target ratio (i.e., most imbalanced). The implicit z‐axis representing total digestible structural carbohydrates (TSC) is not shown. (b) Frequency (indicated by circle size and color) of observed feeding on consecutive pairs of complementary food items, that is, the food item with the higher AP:TNC + lipids ratio in a pair was eaten prior to the one with a lower AP:TNC + lipids ratio or vice versa. For example, the most frequently consumed combination of complementary food items was *Filipendula ulmaria* and *Rumex longifolius*.

The diets as if consisting of only the individually imbalanced food items (i.e., with the balanced food items computationally removed from the data) were also very similar to the macronutritional target (Figure 6a). Moose on average switched 93 times between nutritionally complementary forages during the observation period. Autocorrelation analysis revealed a strong negative autocorrelation at lag 1 (Durbin–Watson tests, moose ID1652: dw = 2.84, *p* < .001; ID1764: dw = 2.69, *p* < .001; IDE2808: dw = 2.46, *p* < .03). This means that complementary feeding was non‐random and followed a ‘zigzag’ pattern where foraging above the AP:TNC + lipids ratio of the macronutritional target was followed by foraging below the target (Figure S3). Simulated random feeding (1000 iterations of 93 switches between complementary foods) on complementary food items was not autocorrelated (dw_mean_ = 2.09, *p*_mean_ = .48). The mean AP:TNC + lipids ratios of observed and simulated complementary feeding did not differ (Welch's *t*‐test, *t*_mean_ = −0.54, *p*_mean_ = .51), but the simulated feeding showed larger variance (Levene's test, *F*_mean_ = 36.51, *p*_mean_ < .007), in line with moose not feeding randomly. Complementary feeding occurred most frequently between *F. ulmaria* and *R. longifolius* (Figure 6b).

Birch was the only deciduous forage where moose fed on stripped leaves and leaves+twigs separately, but there were strong differences between individuals (percentage of leaf stripping when feeding on birch: IDE2808 (96%), ID1764 (86%), and ID1652 (30%); Figure S4a). In deciduous tree forages, leaves and twigs generally had a similar AP:TNC + lipids ratio, whereas in birch, the ratio for twigs was much lower than for leaves (i.e., leaves were closer to the nutritional target ratio, with twigs below the target ratio). *S. aucuparia*, generally considered a highly preferred food item for moose, showed the opposite pattern; with twigs being closer to the target ratio than the leaves, and the leaves being above the target ratio (Figure S4b).

Most forages (including the main food item, *Salix*) were low in sodium content and did not reach the recommended threshold for domestic ruminants of 0.1% DM. Notable exceptions were ley‐mix, *Taraxacum* spp., and *M. trifoliata*. The plant with the highest sodium content, white water‐lily (*Nymphaea alba*) was not consumed during the study period (Figure S5). No forage fell below the critical threshold of 2:1 for the Cu:Mo ratio and most forages (including *Salix*) were above the 5 ppm threshold for copper deficiency (Figure S6). All forages exceeded the suggested minimum concentration of 0.2% DM for calcium. The average diet and *Salix* met the minimum threshold (0.2% DM) for phosphorus, but several forages fell well below it. The diet average and most forages were above the suggested range of 1:1 to 2:1 for the Ca:P ratio but well below the upper threshold of 7:1 (Figure S7). Magnesium concentrations in the eaten plants ranged from 0.08 to 0.55% DM ($\bar{x}$ = 0.24 ± 0.11SD).

## 4 DISCUSSION

Our study is one of the first to give evidence of nutritionally complementary feeding in a free‐ranging northern herbivore during summer, and aligns well with findings for macronutrient balancing in moose during winter (e.g., Spitzer et al. (2023), Felton et al. (2021)). Moose tightly regulated their intake of protein to highly metabolizable macronutrients (AP:TNC + lipids) to a certain ratio. They reached their intake target by consuming large amounts of nutritionally balanced *Salix*, or by combining nutritionally imbalanced foods in a non‐random manner that minimized deviations from the intake target.

### 4.1 Botanical diet composition and food niche

As expected, the botanical diet composition aligned well with the established classification of moose as browsers, as opposed to being a grazer, even in summer. Moose foraged predominantly on deciduous tree species with *Salix* contributing just over 50% to the average diet. The selection for *Salix* we observed has also been shown in previous moose studies (e.g., Cederlund et al. (1980), Shipley et al. (1998)), while the total share of deciduous tree species (71%) was in the upper end of what has been found for moose populations further south in Norway (27–80%, Wam and Hjeljord (2010a, 2010b)).

Despite an abundance of available forages, the realized food niche (both botanically and nutritionally) was relatively small with only 9 food items comprising 95% of the diet and occupying only a small part of the nutrient space demarcated by the measured vegetation (Figures 3 and 4). This suggests that moose may tend toward being more of a food composition specialist than a generalist relative to the diets of other cervids such as red deer (Gebert & Verheyden‐Tixier, 2001) or roe deer (Cederlund et al., 1980). On the other hand, moose still consumed 25 different food items over the relatively short observation period of 5 days and the macronutrient proportions in their diets were not significantly different from the forage plant averages. This suggests also some general adaptation to the nutrient proportions seasonally available in their environment and fits well with the conclusion in Shipley's comprehensive review (Shipley, 2010) that moose are neither food specialists nor generalists in the traditional sense, but facultatively somewhere on the continuum between the two, depending on, for example, location.

### 4.2 Macronutrient balancing

In terms of macronutrient energy, moose had a diet rich in carbohydrates, moderate in protein, and low in fat, as expected from the type of foods they are adapted to eat. Unexpectedly, the observed protein energy to non‐protein energy (PE:NPE) ratio of the focal individuals' diets in July (0.15) was lower than the moose’ nutritional target suggested by a feeding experiment in winter (0.22, Felton et al. (2016)), but still fell within the observed range (0.12–0.41) of that feeding trial. This could imply that moose select more for protein in winter than in summer, contrary to a commonly held belief. Yet, the notion that northern cervids select for energy in winter (for survival), and protein (for growth) in summer has recently been scrutinized in a literature review and found to not be based on evidence (Felton et al., 2018).

Further analysis of our data suggested that the foraging choices were driven in part by macronutrient balancing between protein and the easily digestible macronutrients (TNC + lipids). Available protein and TNC + lipids were the most strongly correlated macronutrients in the diet but were not correlated across forage plants (Table 1). The nutritional rail representing the AP:TNC + lipids ratio for the intake target intercepted *Salix*, indicating *Salix* as a nutritionally balanced food in this regard (Figure 4). A nutritional target resembling *Salix* has also been reported for the winter in free‐ranging moose (Spitzer et al., 2023) and experimental settings (Felton et al., 2016), lending further evidence that *Salix* may be a food resource of particular importance to moose.

Being nutritionally balanced in a certain dimension, however, does not automatically make a food exploitable. For this, the physical and non‐nutritional dimension of the multidimensional nutritional niche framework must also be considered. For example, abundantly available mire graminoids (mostly different sedges) in our study area had similar AP:TNC + lipids ratios as the frequently eaten birch leaves or *E. angustifolium*, but were never observed to be eaten by moose during the study period. This may be because moose rumens are poorly adapted to digest the fibrous structure of graminoids (Clauss et al., 2010). This part of our results therefore also fits well with the view of moose as a food exploitation specialist that is largely restricted to dicots such as tree browse and forbs, that is, a specialized browser or ‘non‐grazer’ (Van Wieren, 1996).

#### 4.2.1 Complementary feeding and the role of Salix

Since *Salix* represented the main food item, it would be circular to argue for intentional nutrient balancing based on the nutritional similarity between *Salix* and the diets. However, we found that diets from which *Salix* and the other balanced food items had been computationally removed, which thus consisted of only nutritionally imbalanced foods, still resulted in average diets with nearly identical macronutrient proportions and AP:TNC + lipids ratio as the full diets (Figure 6). Moose accomplished this by feeding on individually imbalanced but nutritionally complementary food items in a non‐random alternating ‘zigzag’ pattern around the target AP:TNC + lipids ratio (Figure S3). Moreover, simulated random feeding sequences using the same complementary foods showed the same mean AP:TNC + lipids ratio as the observed complementary diets but with significantly higher variation around the target ratio. This strongly suggests that moose intentionally track the AP:TNC + lipids ratio of their diet and avoid large deviations. Further research using controlled feeding trials with feeds spanning a large gradient of AP:TNC ratios should explore how much deviation from the intake target ratio moose are willing to tolerate and which strategy they chose when faced with only imbalanced foods, that is, their ‘rule of compromise’ (Simpson & Raubenheimer, 2012). Lipids probably play only a small role in this interaction as plant tissues were low in lipids. The absence of a positive correlation between the relative availability of forages in the environment and their proportions in the observed diet further supports non‐random feeding choices by moose.

Our findings align well with the results of Felton et al. (2021) who also reported a tight relationship between AP and TNC + lipids in moose rumens across a large variety of winter diets at several locations throughout Sweden. Similarly, Spitzer et al. (2023) showed that moose used complementary feeding on *P. sylvestris* and *Vaccinium* spp. shrubs in winter to attain a protein to total carbohydrate balance, which resembled that of *Salix*. The balancing of protein and TNC simultaneously requires regulatory flexibility of structural carbohydrates (TSC). Since TNC and TSC were negatively correlated in forage plants (Table 1), reaching a target balance between protein and TNC from forages low in TNC concentration would inevitably require moose to ingest more TSC or the other way around. We did not observe a larger variation in TSC intake than in TNC intake across meals, but TNC was consumed in lower amounts than what would have been expected from the forage plants (Figure S2). The similar variation of TSC and TNC intakes may be a result of the relatively few food items consumed which did not span a large gradient in TSC content, whereas the lower‐than‐expected intake of TNC may be an indication of an intrinsic caution against the intake of foods with high TNC content, which can upset the rumen balance (Butler et al., 2008). While it is unlikely that the TNC levels of commonly eaten, naturally occurring forages could dangerously imbalance the rumen pH, selection of lower sugar content by moose has been reported for as fine a scale as different birch trees (Wam et al., 2018). Felton et al. (2021) found a large variation of TSC content in moose rumen, while protein and TNC were tightly regulated. This may, however, have been partly due to the longer retention time in the rumen of the less digestible TSC (Van Soest, 1994). This could lead to greater variation of TSC content in the rumen than could be inferred solely from observations of intake.

Interestingly, the TSC concentration of *Salix*, and the average diet based on the complementary food items, closely matched the TSC content of the intake target, which may be yet another indication of why *Salix* can be a nutritionally balanced food for moose (Figures 4 and 6a). Ruminants depend on a continuous intake of dietary fiber (the TSC components’ cellulose and hemicellulose) to maintain their commensal cellulolytic rumen microorganisms and for the stimulation of acid‐neutralizing saliva secretion (Allen, 1997). Since acid production in the rumen is largely a result of carbohydrate fermentation, balancing sugars and fibers should be important in this context (Keunen et al., 2002) just like the balance between protein and carbohydrates is important for microbial nitrogen metabolism and the detoxification of plant secondary metabolites (Villalba & Provenza, 2005). Felton et al. (2016) suggested that eating large amounts of *Salix* may safeguard moose from dramatic shifts in rumen pH due to the presence of tannins that can bind to proteins and carbohydrates with high molecular weight (Shahidi & Naczk, 1992). While other deciduous species can also have this tannin effect, they may be further from the moose nutritional target. *Salix* also contained suitable concentrations of copper, molybdenum, calcium, and phosphorus but was very low in sodium content (Table S2b), which may be one reason for why *Salix* was not eaten in even higher proportions.

#### 4.2.2 Differential feeding on birch parts – A case of bite‐scale macronutrient balancing?

While diet composition and foraging behavior were very similar across moose individuals, birch presented a notable exception to all other deciduous foods. The proportions of feeding on stripped birch leaves versus leaves and twigs together differed among the individuals (Figure S4a). Two individuals almost exclusively stripped leaves (96 and 86% of feeding observations on birch), while the third most frequently consumed leaves and twigs together (70%). This may present an example of fine‐scale nutrient balancing.

Birch leaves were nutritionally balanced with respect to the AP:TNC + lipids intake target, whereas birch twigs were unbalanced (well below the nutritional rail of the target). This contrast between leaves and twigs in birch was larger than for the other deciduous forages except for *S. aucuparia* (Figure S4b). The best strategy for a nutritionally balanced intake of birch would therefore be to strip leaves, that is, the behavior shown by two of the three moose individuals. Consuming only leaves+twigs together would result in a AP:TNC + lipids ratio below the target, which may explain why the third individual (ID1652), which largely ingested leaves+twigs together when feeding on birch, still sometimes stripped the leaves. Interestingly, this individual also consumed higher amounts of forages that were nutritionally complementary to birch leaves+twigs than the other two moose, and also had a higher proportion of *Salix* in its diet although these differences were not significant (Figure S4a).

*Sorbus aucuparia* was also relatively imbalanced but in an opposite pattern to birch: Here, it was the twigs and not the leaves that were on the target rail (*S. aucuparia* leaves were well above the rail). However, since it is not efficiently possible to consume twigs separately from leaves from deciduous trees during the summer, all moose foraged on *S. aucuparia* by consuming leaves and twigs together just as they did for all other deciduous forages except birch. Notably, Wam and Hjeljord (2010a, 2010b) found that moose in southern Norway selected more for birch in summer than in winter, while the opposite pattern was found for *S. aucuparia*. This would align with our observation of birch foliage and *S. aucuparia* twigs being nutritionally balanced but remains speculative as we did not test how nutrient proportions might change from the summer to the winter.

### 4.3 Minerals

In terms of mineral content, the main forages generally fell into the ranges suggested to be suitable for ruminants, except for sodium, where many forages did not reach the threshold for minimum requirement (Figure S5). Low levels of sodium in moose forages have been well documented (Belovsky & Jordan, 1981; Botkin et al., 1973). Moose typically respond to sodium shortages by eating aquatic macrophytes such as water lilies (Nymphaeaceae), which are high in sodium content (Fraser et al., 1980, 1984). We did not observe any feeding on water lilies, which had the highest sodium content of all analyzed forages. Instead, the three moose frequently fed on *M. trifoliata*, an emergent aquatic perennial which had the second highest sodium content among the sampled vegetation and almost twice the dry matter content (15%) of *N. alba* (8%).

### 4.4 Caveats and limitations

Our results should be considered in the context of some possible limitations. Due to the trade‐off between detailed observations and data storage limitations of the camera collars, the study period of 5 days was relatively short. However, Felton et al. (2016) reported that during a feeding trial with imbalanced foods, homeostatic responses such as the stabilization of protein:non‐protein intake ratios occurred within 30–60 h. Although the sample size of three individuals was small, their macronutrient balancing patterns were still basically identical, whereas they showed inter‐individual differences in foraging choices such as the differential feeding on birch or the different proportions of, for example, *M. trifoliata* or *F. ulmaria* in their diets (Figure 2b).

Despite following slightly different foraging paths, all three moose inhabited the same geographical area during the study (Figure 1). It is therefore difficult to untangle whether the observed similar patterns of nutrient balancing resulted from randomly encountering essentially the same food resources or whether moose actively chose the area precisely because the available forages made it easy to attain a balanced diet. Studies of habitat selection by moose on Vega Island suggest the latter as moose have been shown to select habitats rich in deciduous forests with tall herbs and males preferring open areas with short herbs in the summer over the winter (Ofstad et al., 2019). This aligns well with the characteristics of the study area. Moreover, the GPS positions of 34 additional moose (without cameras) showed that they mostly used the same areas as the three individuals in our study during the observation period (Figure S8). We also frequently observed the presence of such additional moose on the video footage, sometimes resulting in groups of up to ~10 individuals, which is unusually gregarious behavior for moose during the summer and points toward the study area offering highly suitable habitats.

The circumstance that moose on Vega are also among the heaviest and most fecund in Norway (Solberg et al., 2011) further strengthens the notion that part of this suitability is linked to the properties of the available food resources and that resources were sufficient for these moose to be able to be selective (i.e., experienced little food limitation). Access to resources on Vega is also less limited by human infrastructures compared to most mainland moose habitats in Norway. The collared moose frequently crossed pastures and passed within sight of human settlements, which supports previous observations that, while moose in general tend to avoid areas close to humans, and males (all study animals were males) are more willing to use areas near houses (Lykkja et al., 2009; Mehlhoop et al., 2022). Thus, the Vega moose were foraging under near ideal conditions compared to many other moose populations.

Future research should track nutrient balancing across all seasons in a wider variety of habitats and also include females and calves. Only then will we gain a full understanding of where moose fit along the gradient from nutritional specialist to generalists. Longer‐term studies are needed to untangle how macronutrient balancing, forage quantity, and quality interact in their effects on the condition and fitness of moose and other cervids. In this context, the role of the host–rumen microbiota interactions deserve special attention. Methods for plant nutrient analyses should also be standardized to facilitate direct comparisons across studies.

## 5 CONCLUSION

Overall, our analysis thus suggests that moose showed macronutrient balancing by strictly regulating their intake of protein to highly digestible macronutrients (AP:TNC + lipids). They reached their intake target by consuming nutritionally balanced forages (primarily *Salix*) or through combining nutritionally complementary foods in a non‐random manner that minimized deviations from the macronutrient ratio of the intake target. Differential feeding on birch suggested that macronutrient balancing may occur on as fine a scale as foraging bites on a single food item. The patterns of macronutrient balancing we observed during the summer aligned well with the findings of other studies conducted during the winter.

## AUTHOR CONTRIBUTIONS

**Robert Spitzer:** Conceptualization (equal); data curation (lead); formal analysis (lead); funding acquisition (equal); investigation (equal); methodology (equal); project administration (lead); visualization (lead); writing – original draft (lead); writing – review and editing (equal). **Monica Ericson:** Conceptualization (supporting); data curation (supporting); formal analysis (supporting); funding acquisition (supporting); investigation (supporting); methodology (supporting); writing – review and editing (equal). **Annika M. Felton:** Conceptualization (equal); formal analysis (supporting); methodology (equal); writing – review and editing (equal). **Morten Heim:** Conceptualization (supporting); data curation (supporting); methodology (supporting); project administration (supporting); resources (equal); writing – review and editing (supporting). **David Raubenheimer:** Formal analysis (supporting); methodology (equal); writing – review and editing (equal). **Erling J. Solberg:** Conceptualization (equal); funding acquisition (equal); methodology (supporting); project administration (supporting); writing – review and editing (equal). **Hilde K. Wam:** Writing – review and editing (equal). **Christer M. Rolandsen:** Conceptualization (equal); funding acquisition (equal); methodology (equal); project administration (supporting); writing – review and editing (equal).

## FUNDING INFORMATION

RS was supported by grants from Naturvårdsverket (SEPA; 2020–00108) and Svenska Jägareförbundet (5909/2021). EJS, CMR, and MH were funded by the Norwegian Environment Agency and the Norwegian Institute for Nature Research (NINA). ME was supported by a grant from Stiftelsen Fonden till Tor Jonssons minne.

## CONFLICT OF INTEREST STATEMENT

The authors declare no competing interests.

## Supporting information


Data S1.

## Data Availability

The data that support the findings of this study are openly available in the Dryad repository at: doi:10.5061/dryad.08kprr598.
