# Supplementary material for: Camera collars reveal macronutrient balancing in free‐ranging male moose during summer
Source: Ecol Evol. 2024 Aug 16;14(8):e70192. doi: 10.1002/ece3.70192 (PMC11329299; doi:10.1002/ece3.70192)
Supplement: Supplementary file 1 — Data S1. [file ECE3-14-e70192-s001.pdf]

Supporting Information

Camera collars reveal macronutrient balancing in free-ranging male moose during summer

FIGURES:

- Fig. S1 Average proportions of behaviors states across 24h
- Fig. S2 Proportions of plants components across diets and forage plants
- Fig. S3 Sequences of complementary feeding on imbalanced food items
- Fig. S4 (a) Differential birch browsing, (b) RMT of leaves vs. twigs for decid. browse
- Fig. S5 Bar plot of sodium (Na) concentrations in vegetation samples
- Fig. S6 Scatterplot of copper (Cu) and molybdenum (Mo) concentrations
- Fig. S7 Scatterplot of calcium (Ca) and phosphorus (P) concentrations
- Fig. S8 Map of Vega Island showing the areas most frequented by moose

TABLES:

- Tab. S1 Nutrient and mineral analyses
- Tab. S2a Nutritional composition of moose forages
- Tab. S2b Mineral content of moose forages

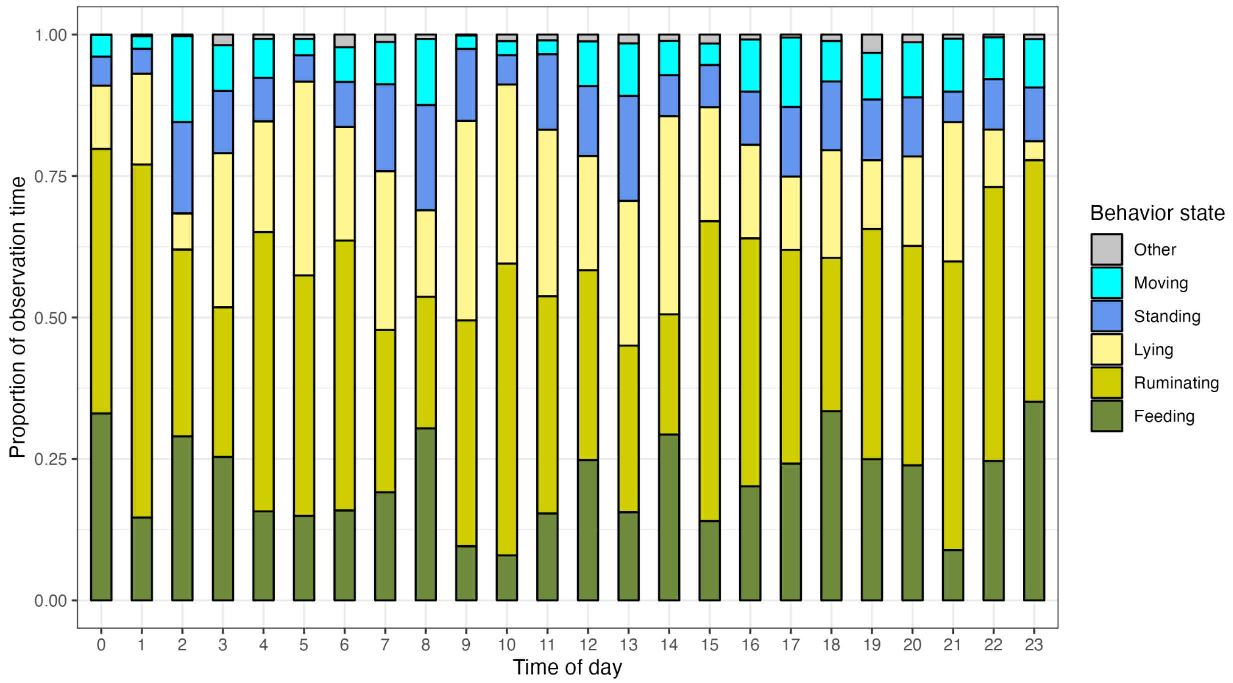

Fig. S1 Average proportion of observation time for different behavior states of moose (N=3) during the different hours of the day during the 5-day observation period on Vega Island (Norway) in July 2022.

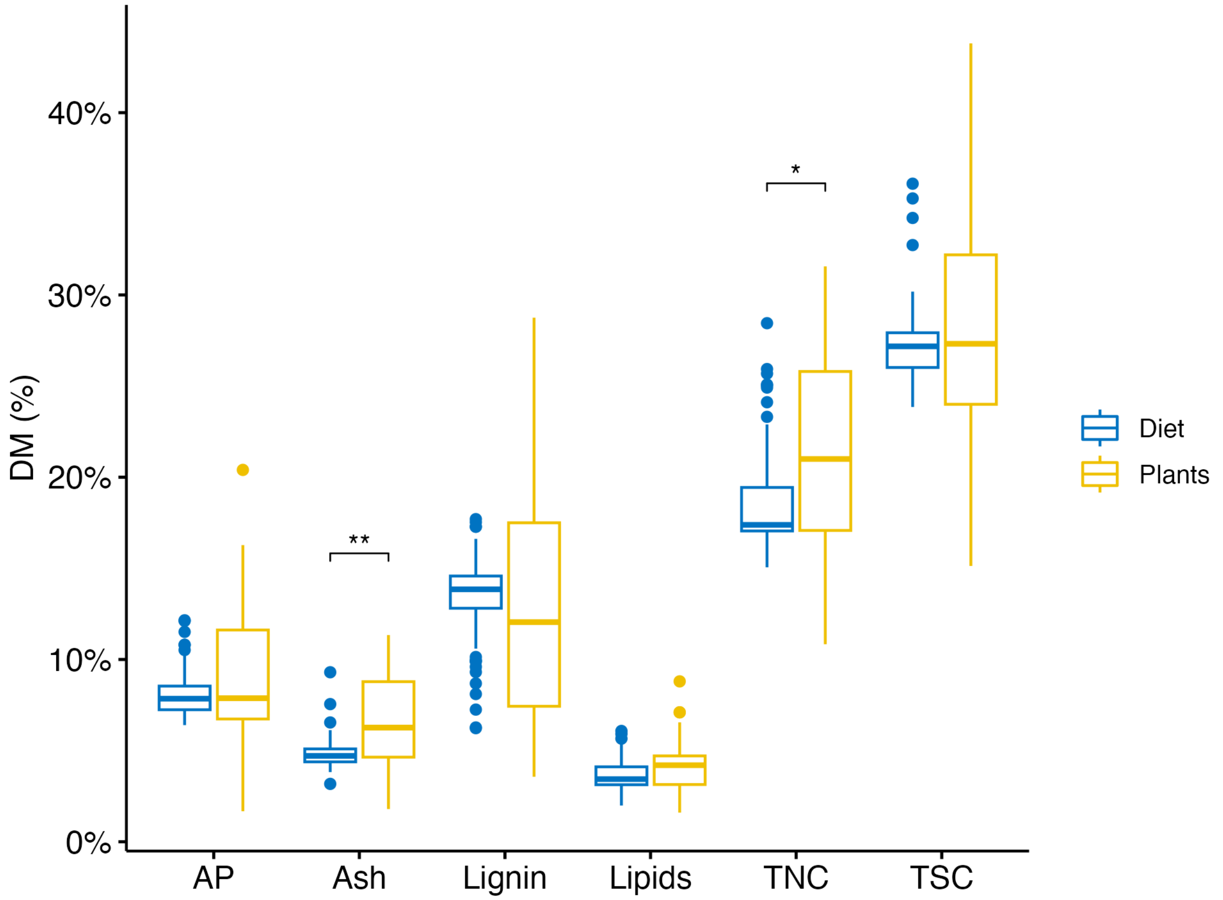

Fig. S2 Boxplot showing the dry-matter (DM) proportions of macronutrients (AP: available protein, TNC: total non-structural carbohydrates, TSC: total structural carbohydrates, and lipids) and other plant components in moose diets (as proportionally eaten, blue) and across 25 eaten forages (as if moose had fed on them in random proportions, yellow) on Vega Island (Norway) in July 2022. Significant differences are indicated by asterisks (Welch's t-test, $*p < 0.05$, $**p < 0.01$).

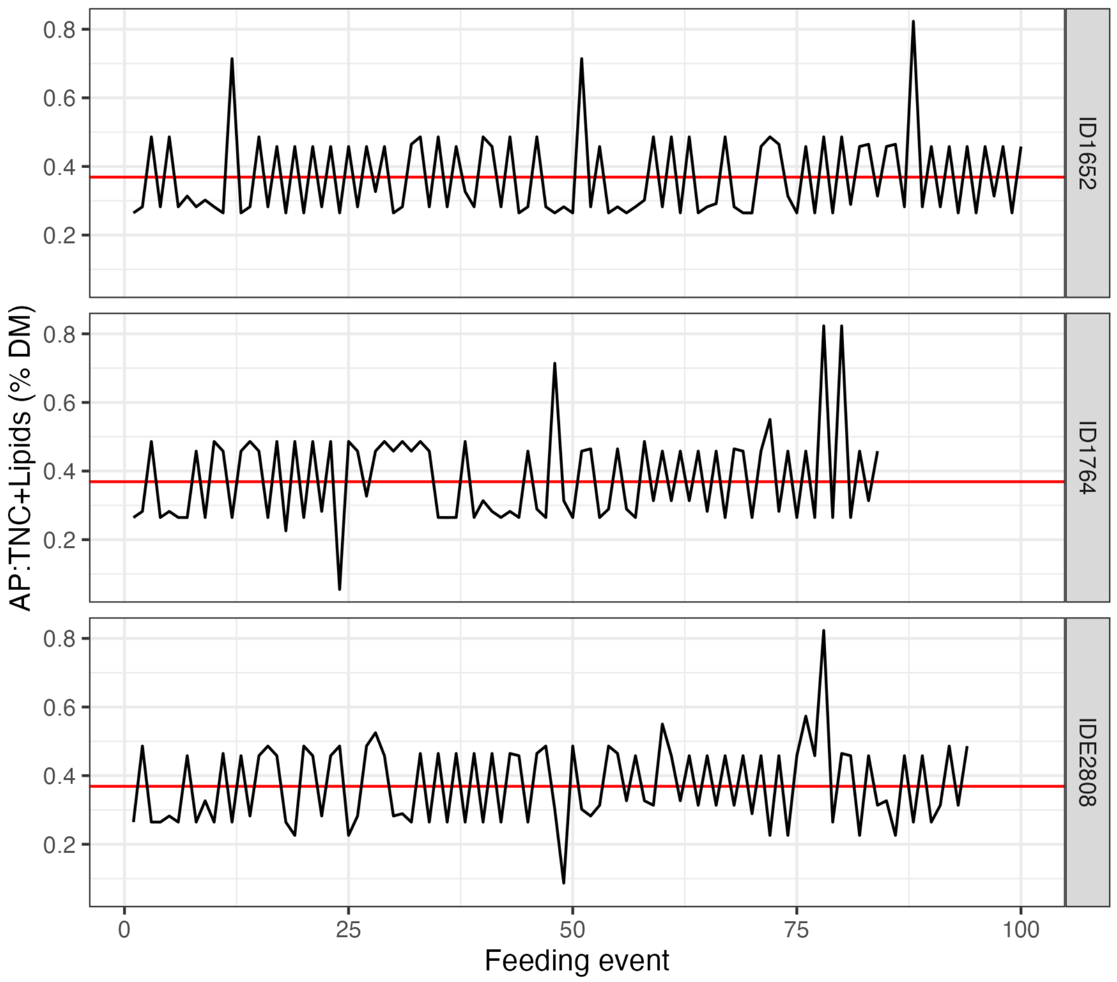

Fig. S3 AP:TNC+lipids ratios of consecutive feeding events by three moose (ID1652, ID1764, IDE2808 on Vega Island, Norway July 2022) on nutritionally imbalanced (complementary) food items in relation to the AP:TNC+lipids ratio of the intake target (0.37, red line).

a)

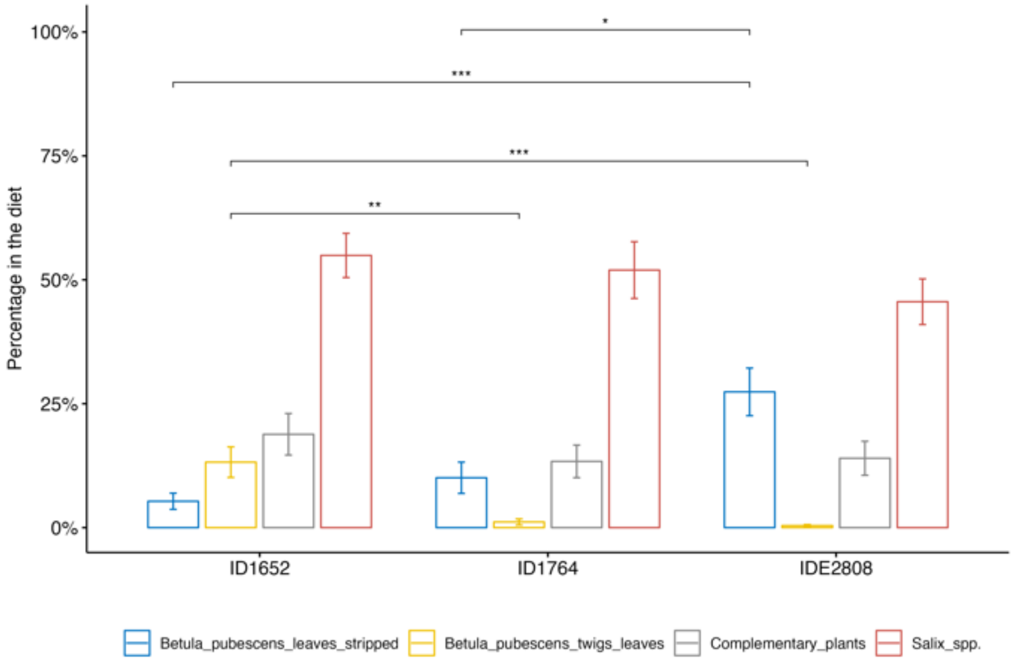

b)

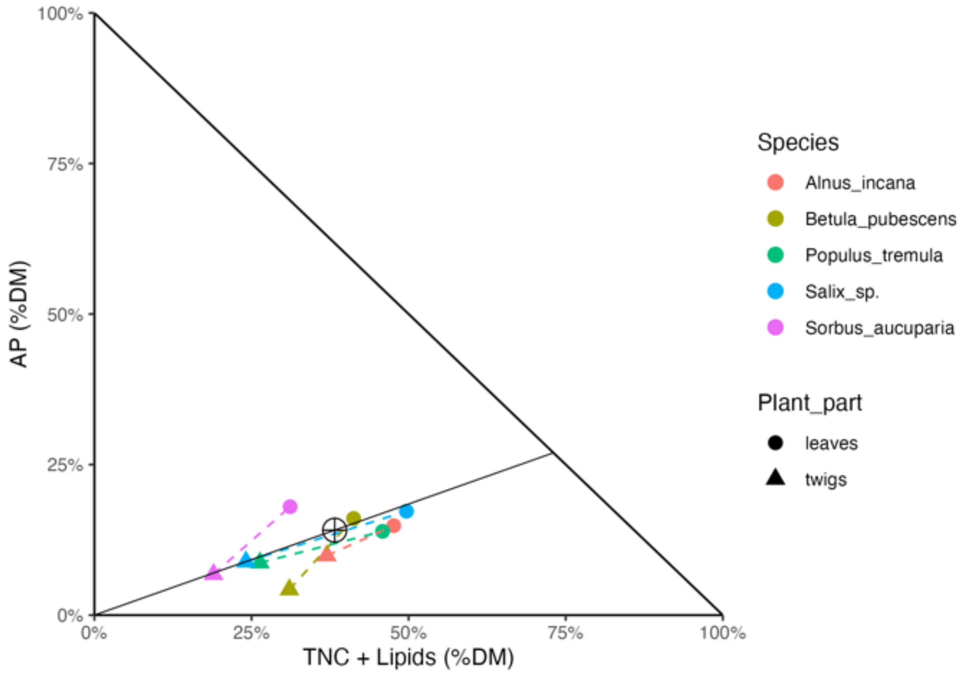

Fig. S4 (a) Mean proportions of birch (*B. pubescens*) parts (stripped leaves and leaves+twigs), *Salix*, and food items that were nutritionally complementary to birch leaves+twigs in the diet of three moose individuals (ID1652 - IDE2808) on Vega Island, Norway in July 2022. Error bars indicate the standard error. The proportions of birch leaves

and twigs+leaves in the diet differed across the individuals. IDE2808 predominantly stripped leaves whereas ID1652 mostly consumed twigs and leaves together. Differences between means were tested using t-tests with Bonferroni corrections of p-values and are denoted with asterisks (*p < 0.05, **p < 0.001, ***p < 0.0001). (b) Right-angled mixture triangle showing the relative components of macronutrient content (AP = available protein, TNC = total non-structural carbohydrates) in moose diets as percentages of total macronutrients in g dry matter (DM). The implicit axis corresponds to total digestible carbohydrates (TSC, cellulose + hemicellulose). The crosshair point indicates the intake target and the intersecting radial (black line) corresponds to the target AP:TNC+lipids ratio. The macronutritional properties of the deciduous forages eaten by moose are plotted in relation to the intake target with colors denoting different plant species and shapes indicating the different plant parts (circles = leaves, triangles = twigs). Except for birch and rowan (*S. aucuparia*), the leaves and twigs of the other deciduous forages were similarly balanced with respect to the ratio of the intake target.

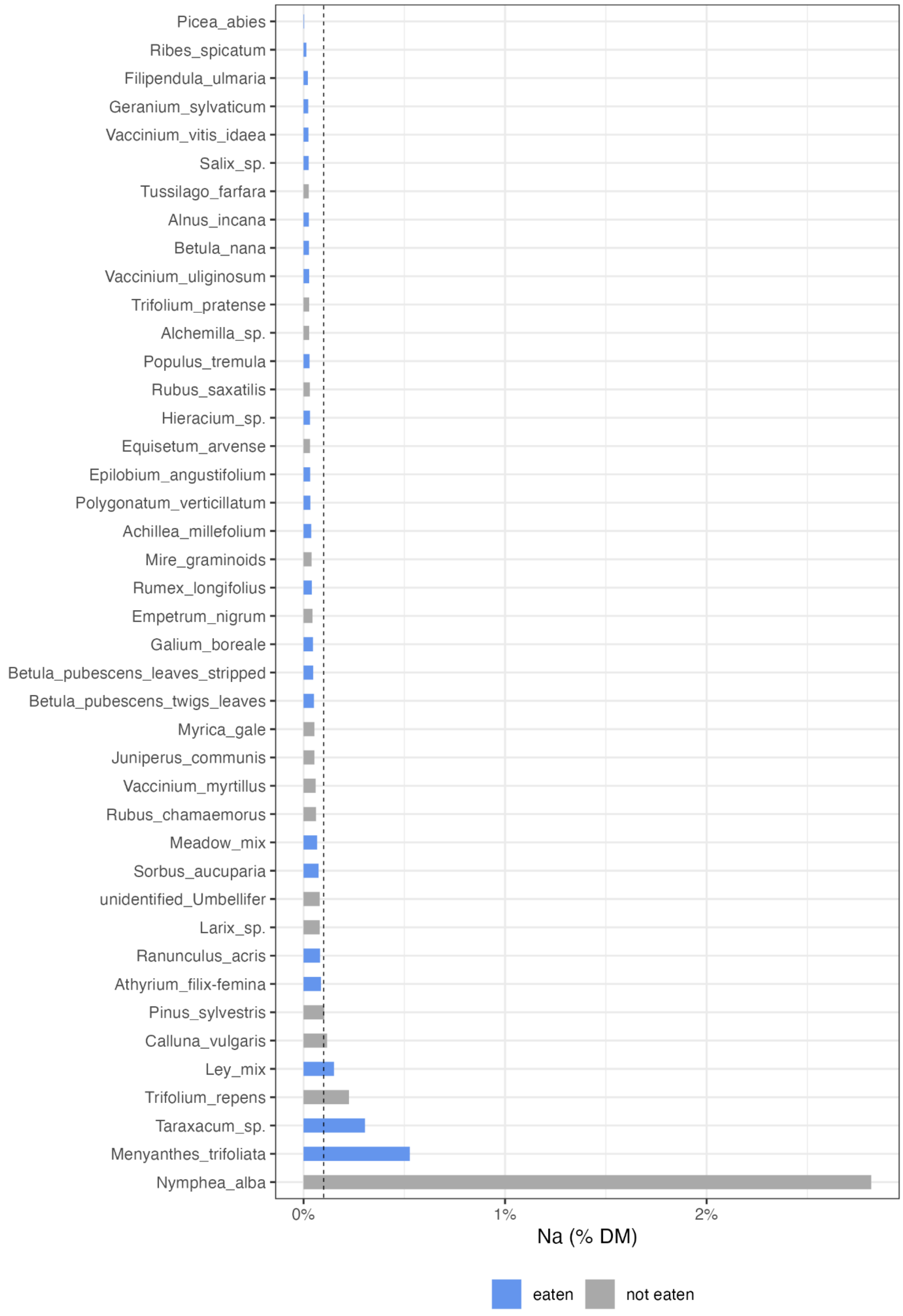

Fig. S5 Sodium (Na) concentrations as percentage of dry matter (%DM) of 42 potential food items for moose collected on Vega Island, Norway during July 2022. For the items in blue, feeding by moose was observed during the observation period. The dashed vertical line indicates the suggested minimum threshold for sodium (0.1% DM) in ruminant forages.

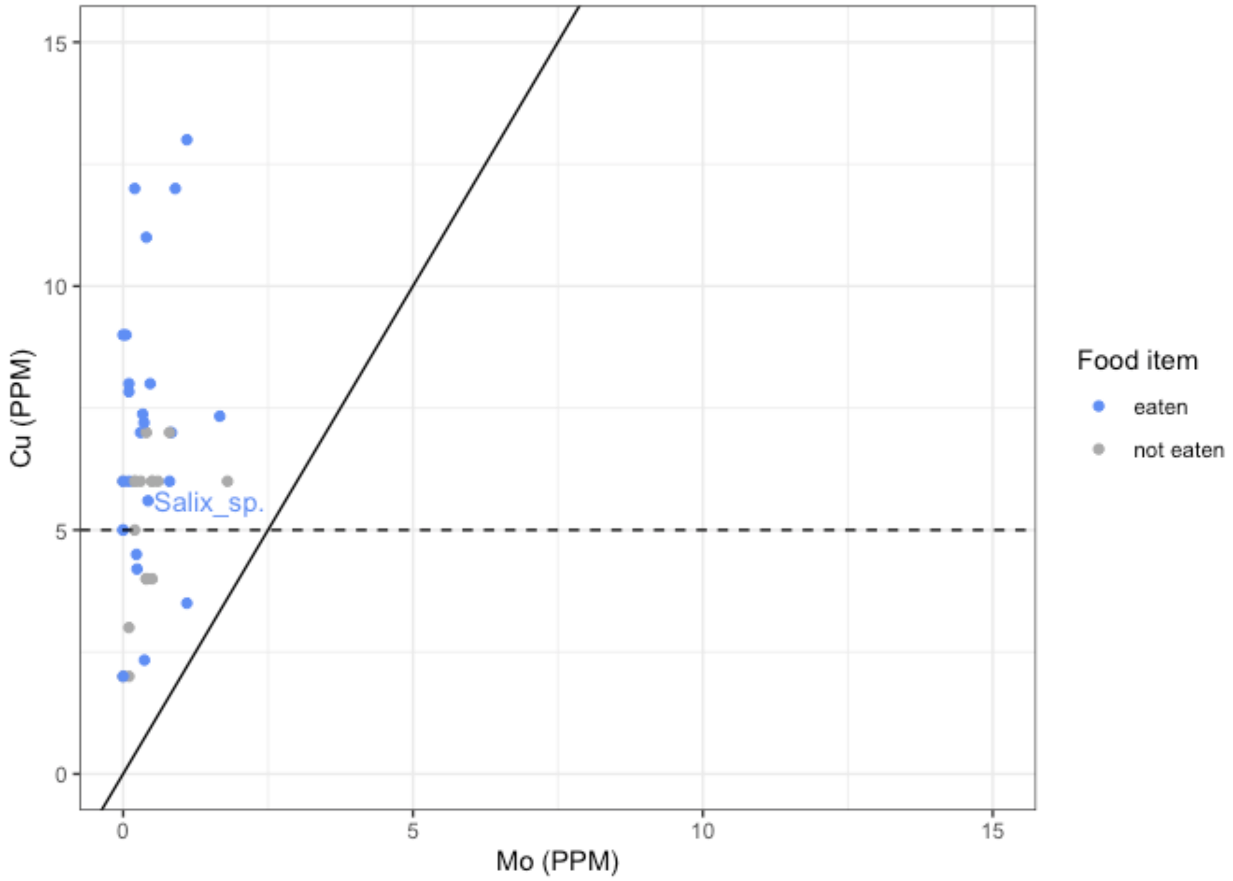

Fig. S6 Scatterplot of molybdenum (Mo) and copper (Cu) concentrations in vegetation samples from Vega Island, Norway July 2022 (blue = feeding by moose observed during the study period) with the thresholds for Cu deficiency (dashed horizontal line) and the minimum suggested Cu:Mo ratio (2:1, solid line).

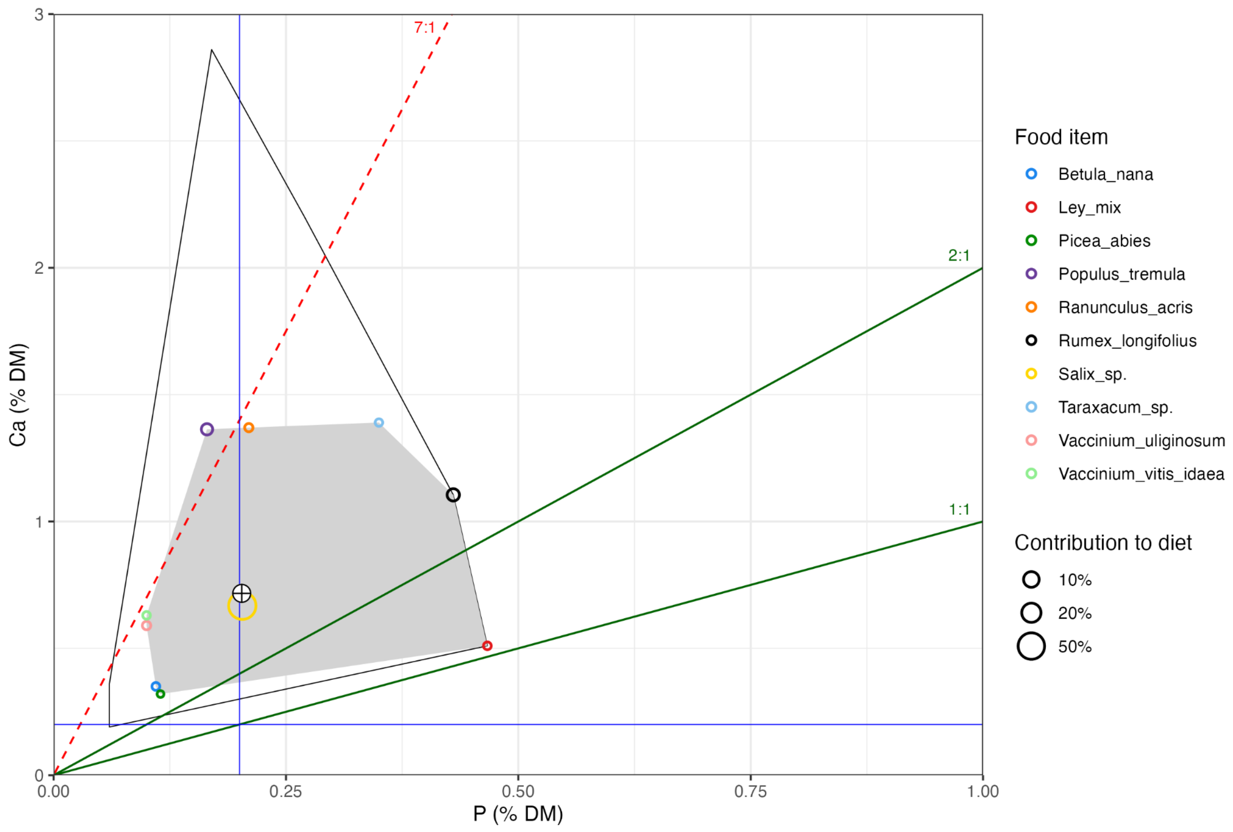

Fig. S7 Scatterplot of calcium (Ca) and phosphorus (P) concentrations in 42 putative moose forages on Vega Island, Norway in July 2022. The black polygon demarcates the outer hull of the concentrations across all forages whereas the gray polygon indicates the niche realized by moose (i.e., the forages that were eaten; N=25). For the latter, the food items forming the corners of the realized niche are shown in different colors with the size of the circles corresponding to their percentage contribution to the average moose diet. Additionally, *Salix* (the main food item) is shown in yellow together with the Ca and P concentrations in the average diet (crosshair point). Radials in green indicate the suggested optimal range of the Ca:P ratio for ruminants whereas the dashed red radial indicates the upper threshold. The thin blue lines correspond to the suggested minimum concentrations of Ca and P required by ruminants.

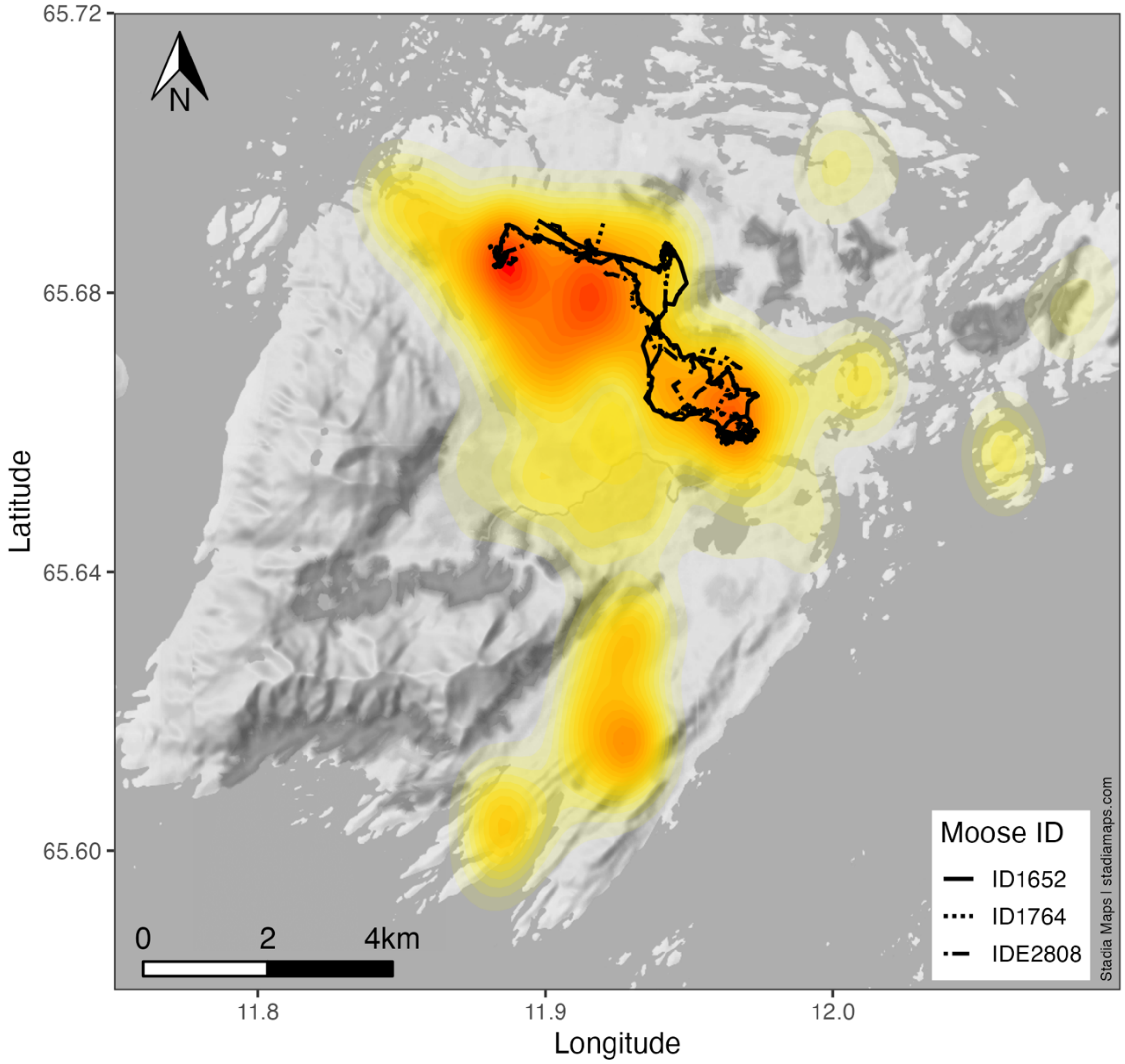

Fig. S8 Map of the study area on Vega Island, Norway. The routes walked by the three camera collared moose during the 5-day observation period (1 to 5 July 2022) are shown as different line types. The density isopleths highlight the data distribution of GPS positions from 34 additional moose (without cameras) between 30 June to 6 July, with shading towards darker tones (red) corresponding to higher density, i.e. indicating the areas most frequented by moose during the study period (GPS data from https://www.dyreposisjoner.no, accessed on 23-05-2024, 8 GPS location fixes per individual and day).

Table S1 The nutritional properties and analytic approach used to study forage plants for moose collected on Vega Island, Norway July 2022. The numbers in square brackets refer to the service package numbers of the DairyOne Forage Laboratory (USA) where the analyses were performed. Detailed methodological descriptions of the chemical analyses used by the laboratory are available at https://dairyone.com/download/forage-forage-lab-analytical-procedures.

**Property**	**Unit**
Acid detergent fiber (ADF) [22]	% DM
Acid detergent insoluble crude protein (ADICP) [30]	% DM
Neutral detergent fiber (aNDF) [23]	% DM
Fat (Lipids), ether extract [126]	% DM
Lignin [28]	% DM
Starch [34]	% DM
Total ash [26]	% DM
Total Nitrogen (N) [20]	% DM
Water soluble carbohydrates (WSC) [254]	% DM
Calcium [41]	% DM
Phosphorus [42]	% DM
Magnesium [43]	% DM
Potassium [44]	% DM
Sodium [45]	% DM
Iron [46]	PPM
Zink [47]	PPM
Copper [48]	PPM
Manganese [49]	PPM
Molybdenum [50]	PPM

Table S2a Nutritional composition 42 putative moose forages collected on Vega Island (Norway) in July 2022. Values are given as percentages of dry matter (%DM). For items that were consumed by moose during the 5-day observation period, their contributions to average moose diets are given as percentages. For the deciduous forages, the proportions in the diets correspond to twigs and leaves together.

				Wet chemistry									Calculated				
**Species**	**Diet (%)**	**Plant_part**	**DM (%)**	**Total N**	**ADICP**	**ADF**	**NDF**	**Lignin**	**Starch**	**WSC**	**Lipids**	**Ash**	**CP**	**AP**	**Cell**	**HCell**	**TNC**
Achillea millefolium	0.12	whole_plant	22.45	2.40	4.00	35.40	49.50	11.70	1.00	10.70	3.70	8.93	15.00	11.00	23.70	14.10	11.70
Alchemilla sp.		whole_plant	22.58	1.40	1.40	19.20	25.70	2.60	6.80	26.70	3.50	8.00	8.75	7.35	16.60	6.50	33.50
Alnus incana	0.01	leaves	34.28	2.80	10.30	47.10	51.60	33.40	4.40	14.00	4.70	5.59	17.50	7.20	13.70	4.50	18.40
		twigs	30.82	1.50	4.30	45.30	51.40	24.10	1.40	14.60	3.00	3.28	9.38	5.08	21.20	6.10	16.00
Athyrium filix-femina	0.45	whole_plant	27.38	1.95	6.00	30.85	40.90	14.70	14.55	11.25	1.60	5.40	12.19	6.19	16.15	10.05	25.80
Betula nana	0.10	twigs_leaves	45.68	2.00	4.80	29.30	40.80	17.90	3.10	15.30	7.10	5.23	12.50	7.70	11.40	11.50	18.40
Betula pubescens	14.26	leaves	39.16	2.08	3.98	22.22	36.38	12.38	4.20	13.06	5.92	4.01	13.00	9.02	9.84	14.16	17.26
		twigs	46.27	0.86	3.06	49.68	60.98	25.60	3.62	9.84	3.50	1.75	5.38	2.32	24.08	11.30	13.46
Calluna vulgaris		twigs_leaves	46.12	1.00	3.40	40.60	54.20	25.50	2.30	10.30	8.20	2.25	6.25	2.85	15.10	13.60	12.60
Empetrum nigrum		twigs_leaves	40.66	1.10	2.40	28.00	34.80	17.90	8.00	21.40	15.00	2.70	6.88	4.48	10.10	6.80	29.40
Epilobium angustifolium	0.41	whole_plant	23.62	2.30	0.93	13.73	18.70	3.57	4.43	27.13	3.20	8.83	14.38	13.44	10.17	4.97	31.57
Equisetum arvense		whole_plant	24.00	2.50	3.00	27.30	42.30	5.10	1.80	16.10	4.00	13.36	15.63	12.63	22.20	15.00	17.90
Filipendula ulmaria	11.80	whole_plant	29.07	2.30	1.54	20.58	29.20	5.00	6.52	19.68	1.82	5.52	14.38	12.84	15.58	8.62	26.20
Galium boreale	0.03	whole_plant	29.76	2.80	5.00	33.40	46.60	17.50	1.90	12.80	7.10	7.49	17.50	12.50	15.90	13.20	14.70
Geranium sylvaticum	0.11	whole_plant	24.33	1.90	0.70	22.80	27.80	4.90	2.70	28.60	2.90	8.91	11.88	11.18	17.90	5.00	31.30
Hieracium sp.	0.10	whole_plant	17.57	1.90	4.00	35.00	49.50	9.50	1.30	18.50	4.30	8.54	11.88	7.88	25.50	14.50	19.80
Juniperus communis		twigs_leaves	37.41	1.50	2.00	36.20	46.10	13.50	5.30	10.40	8.80	3.60	9.38	7.38	22.70	9.90	15.70
Larix sp.		twigs_leaves	38.03	1.90	3.80	35.90	46.50	19.30	3.10	11.40	5.20	2.91	11.88	8.08	16.60	10.60	14.50
Ley mix	0.26	whole_plant	15.36	3.77	2.17	25.93	48.23	4.07	2.43	18.83	4.70	9.20	23.54	21.38	21.87	22.30	21.27
Meadow mix	3.24	whole_plant	24.92	2.20	2.13	30.97	49.57	7.43	2.80	18.27	3.93	6.26	13.75	11.62	23.53	18.60	21.07
Menyanthes trifoliata	2.42	whole_plant	14.74	2.60	8.60	33.27	43.03	17.70	1.10	24.83	2.97	9.30	16.25	7.65	15.57	9.77	25.93
Mire graminoids		whole_plant	38.89	1.60	2.90	30.60	59.60	4.30	1.80	13.60	3.50	4.50	10.00	7.10	26.30	29.00	15.40
Myrica gale		twigs_leaves	38.70	2.30	6.10	22.80	30.10	12.20	6.10	26.20	4.30	4.52	14.38	8.28	10.60	7.30	32.30
Nymphea alba		whole_plant	7.97	2.70	2.60	19.90	35.70	3.40	3.30	14.30	3.20	13.23	16.88	14.28	16.50	15.80	17.60
Picea abies	0.01	twigs_leaves	42.36	0.50	1.45	39.90	46.30	18.95	10.10	13.95	6.55	1.80	3.13	1.68	20.95	6.40	24.05
Pinus sylvestris		twigs_leaves	38.58	0.80	1.40	40.10	52.90	13.80	10.40	7.10	7.10	1.61	5.00	3.60	26.30	12.80	17.50
Polygonatum verticillatum	0.05	whole_plant	20.43	2.70	0.60	18.50	22.70	4.90	0.30	21.90	8.80	9.21	16.88	16.28	13.60	4.20	22.20
Populus tremula	2.20	leaves	38.62	2.30	6.67	32.40	41.80	19.50	3.17	15.60	6.63	6.97	14.38	7.71	12.90	9.40	18.77
		twigs	34.10	1.37	3.40	41.87	53.60	15.20	1.33	9.83	4.40	5.28	8.54	5.14	26.67	11.73	11.17
Ranunculus acris	0.39	whole_plant	21.22	1.50	1.80	37.30	52.20	8.40	4.10	17.80	4.30	6.81	9.38	7.58	28.90	14.90	21.90
Ribes spicatum	0.11	twigs_leaves	29.62	2.70	3.50	20.30	26.50	7.40	4.60	16.40	3.30	6.39	16.88	13.38	12.90	6.20	21.00

Rubus chamaemorus		whole_plant	30.47	2.00	3.80	18.30	31.00	5.10	3.30	36.70	4.20	8.55	12.50	8.70	13.20	12.70	40.00
Rubus saxatilis		whole_plant	24.06	1.60	0.60	25.20	35.50	3.80	1.90	12.90	3.70	6.67	10.00	9.40	21.40	10.30	14.80
Rumex longifolius	9.01	leaves	12.88	5.30	3.40	19.20	30.20	7.40	10.30	8.60	3.60	7.35	33.13	29.73	11.80	11.00	18.90
		whole_plant	18.28	1.95	4.00	31.45	39.50	10.65	3.80	24.65	2.50	6.55	12.19	8.19	20.80	8.05	28.45
Salix spp.	51.26	leaves	33.77	2.48	6.00	24.22	31.78	13.56	6.88	16.64	3.82	5.20	15.50	9.50	10.66	7.56	23.52
		twigs	31.01	1.26	3.06	42.94	52.28	15.86	1.74	8.90	2.46	4.09	7.88	4.82	27.08	9.34	10.64
Sorbus aucuparia	3.16	leaves	32.39	2.15	4.10	25.03	38.28	11.90	2.85	9.23	4.08	7.06	13.44	9.34	13.13	13.25	12.08
		twigs	32.76	0.93	1.65	42.63	57.45	12.20	2.73	6.88	1.95	3.96	5.78	4.13	30.43	14.83	9.60
Taraxacum sp.	0.16	whole_plant	14.52	2.50	5.00	25.20	35.00	9.70	1.50	22.50	4.30	11.34	15.63	10.63	15.50	9.80	24.00
Trifolium pratense		whole_plant	22.60	2.50	2.10	34.00	45.90	9.30	2.30	11.30	3.50	7.60	15.63	13.53	24.70	11.90	13.60
Trifolium repens		whole_plant	19.32	2.30	2.30	37.70	47.00	10.50	2.40	14.00	2.90	6.99	14.38	12.08	27.20	9.30	16.40
Tussilago farfara		whole_plant	14.07	2.50	4.00	25.50	36.60	9.70	5.40	9.60	3.00	15.57	15.63	11.63	15.80	11.10	15.00
unidentified Umbillifer		whole_plant	27.22	1.80	2.40	39.30	54.00	13.40	1.50	10.40	8.40	9.44	11.25	8.85	25.90	14.70	11.90
Vaccinium myrtillus		twigs_leaves	40.84	1.20	2.90	35.20	45.10	15.50	3.90	22.80	2.70	3.51	7.50	4.60	19.70	9.90	26.70
Vaccinium uliginosum	0.28	twigs_leaves	39.27	1.70	3.20	37.00	49.10	16.90	2.70	13.30	4.20	2.49	10.63	7.43	20.10	12.10	16.00
Vaccinium vitis-idaea	0.08	twigs_leaves	42.77	1.20	4.80	39.10	47.30	23.10	6.10	20.50	4.50	2.68	7.50	2.70	16.00	8.20	26.60

Table S2b Mineral content of 42 putative moose forages collected on Vega Island (Norway) in July 2022. Values are given as percentages of dry matter (%DM), except for Fe, Zn, Cu, Mn, and Mo, which are given as parts per million (PPM).

		Minerals									
**Species**	**Plant_part**	**Ca**	**P**	**Mg**	**K**	**Na**	**Fe**	**Zn**	**Cu**	**Mn**	**Mo**
Achillea millefolium	whole_plant	1.01	0.39	0.25	2.95	0.04	200.00	40.00	11.00	59.00	0.40
Alchemilla sp.	whole_plant	1.63	0.26	0.26	1.83	0.03	68.00	28.00	4.00	149.00	0.50
Alnus incana	leaves	1.47	0.20	0.21	0.88	0.01	49.00	20.00	9.00	51.00	0.00
	twigs	0.71	0.14	0.13	0.86	0.04	43.00	18.00	9.00	29.00	0.10
Athyrium filix-femina	whole_plant	0.42	0.21	0.38	1.86	0.09	43.00	16.50	7.00	39.50	0.30
Betula nana	twigs_leaves	0.35	0.11	0.16	0.49	0.03	57.00	117.00	5.00	626.00	0.00
Betula pubescens	leaves	0.69	0.15	0.30	0.58	0.05	51.80	149.60	4.20	498.60	0.24
	twigs	0.37	0.08	0.10	0.33	0.05	37.80	116.60	4.80	147.20	0.22
Calluna vulgaris	twigs_leaves	0.36	0.06	0.12	0.46	0.12	44.00	15.00	7.00	599.00	0.40
Empetrum nigrum	twigs_leaves	0.55	0.12	0.15	0.49	0.04	29.00	13.00	6.00	344.00	0.20
Epilobium angustifolium	whole_plant	1.09	0.31	0.28	1.61	0.03	80.67	27.00	8.00	47.33	0.47
Equisetum arvense	whole_plant	2.86	0.17	0.25	2.29	0.03	93.00	26.00	6.00	37.00	0.50
Filipendula ulmaria	whole_plant	0.59	0.22	0.37	1.41	0.02	61.00	38.80	7.20	164.00	0.36
Galium boreale	whole_plant	1.09	0.25	0.23	2.34	0.05	55.00	46.00	8.00	62.00	0.10
Geranium sylvaticum	whole_plant	1.35	0.23	0.27	1.39	0.02	56.00	20.00	6.00	41.00	0.10
Hieracium sp.	whole_plant	0.97	0.24	0.23	2.93	0.03	93.00	31.00	12.00	60.00	0.20
Juniperus communis	twigs_leaves	0.98	0.16	0.10	0.64	0.05	35.00	22.00	5.00	186.00	0.20
Larix sp.	twigs_leaves	0.43	0.14	0.15	0.75	0.08	51.00	22.00	6.00	198.00	0.30
Ley mix	whole_plant	0.51	0.47	0.26	3.02	0.15	291.00	39.67	7.33	105.67	1.67
Meadow mix	whole_plant	0.94	0.23	0.27	1.72	0.07	65.00	37.00	7.00	163.67	0.83
Menyanthes trifoliata	whole_plant	1.07	0.28	0.23	2.51	0.53	380.33	44.33	2.33	442.00	0.37
Mire graminoids	whole_plant	0.62	0.09	0.09	1.17	0.04	69.00	32.00	4.00	132.00	0.40
Myrica gale	twigs_leaves	0.26	0.11	0.08	0.88	0.05	39.00	24.00	4.00	338.00	0.40
Nymphea alba	whole_plant	1.18	0.29	0.16	1.39	2.82	525.00	31.00	2.00	499.00	0.00
Picea abies	twigs_leaves	0.32	0.12	0.08	0.43	0.00	44.00	37.50	2.00	378.50	0.00
Pinus sylvestris	twigs_leaves	0.19	0.06	0.08	0.41	0.10	49.00	23.00	2.00	158.00	0.10
Polygonatum verticillatum	whole_plant	0.98	0.18	0.16	3.34	0.03	43.00	20.00	5.00	40.00	0.00
Populus tremula	leaves	1.74	0.18	0.27	1.11	0.02	43.67	126.67	6.67	103.00	0.13
	twigs	0.99	0.15	0.15	1.16	0.04	29.67	59.33	9.00	25.67	0.07
Ranunculus acris	whole_plant	1.37	0.21	0.16	1.75	0.08	32.00	31.00	12.00	39.00	0.90
Ribes spicatum	twigs_leaves	0.76	0.31	0.21	2.20	0.01	41.00	15.00	6.00	81.00	0.80
Rubus chamaemorus	whole_plant	0.60	0.09	0.55	1.17	0.06	80.00	56.00	3.00	38.00	0.10
Rubus saxatilis	whole_plant	1.16	0.17	0.55	1.72	0.03	42.00	32.00	6.00	69.00	0.20
Rumex longifolius	leaves	0.61	0.39	0.51	2.50	0.05	73.00	25.00	5.00	92.00	0.60
	whole_plant	1.11	0.43	0.31	1.47	0.04	65.50	20.00	3.50	93.00	1.10
Salix spp.	leaves	0.74	0.24	0.23	1.14	0.02	51.80	111.80	4.80	240.00	0.58
	twigs	0.60	0.16	0.12	1.01	0.03	30.20	99.20	6.40	143.00	0.28
Sorbus aucuparia	leaves	1.19	0.21	0.38	1.53	0.05	73.50	18.00	7.75	151.00	0.45
	twigs	0.85	0.12	0.17	0.85	0.10	44.50	36.75	7.00	52.75	0.23
Taraxacum sp.	whole_plant	1.39	0.35	0.52	3.47	0.31	49.00	38.00	13.00	116.00	1.10
Trifolium pratense	whole_plant	1.82	0.21	0.26	1.49	0.03	46.00	29.00	6.00	34.00	0.60
Trifolium repens	whole_plant	0.95	0.26	0.19	2.05	0.23	52.00	18.00	6.00	28.00	1.80
Tussilago farfara	whole_plant	2.20	0.27	0.29	3.84	0.03	325.00	25.00	7.00	49.00	0.80
unidentified Umbillifer	whole_plant	2.03	0.25	0.33	2.09	0.08	93.00	24.00	6.00	71.00	0.50

Vaccinium myrtillus	twigs_leaves	0.80	0.10	0.20	0.64	0.06	38.00	29.00	6.00	747.00	0.00
Vaccinium uliginosum	twigs_leaves	0.59	0.10	0.18	0.40	0.03	34.00	58.00	9.00	1710.00	0.00
Vaccinium vitis-idaea	twigs_leaves	0.63	0.10	0.17	0.44	0.02	27.00	32.00	6.00	524.00	0.00
